# The APMAP interactome reveals new modulators of APP processing and beta-amyloid production that are altered in Alzheimer’s disease

**DOI:** 10.1186/s40478-019-0660-3

**Published:** 2019-01-31

**Authors:** Hermeto Gerber, Sebastien Mosser, Benjamin Boury-Jamot, Michael Stumpe, Alessandra Piersigilli, Christine Goepfert, Joern Dengjel, Urs Albrecht, Fulvio Magara, Patrick C. Fraering

**Affiliations:** 1 Foundation Eclosion, CH-1228 Plan-les-Ouates, Switzerland; 2 Campus Biotech Innovation Park, CH-1202 Geneva, Switzerland; 3 Department of Biology, University of Fribourg, CH-1700 Fribourg, Switzerland; 4 Centre for Psychiatric Neuroscience, Department of Psychiatry, Lausanne University Hospital, CH-1015 Lausanne, Switzerland; 5 Institute of Animal Pathology, Vetsuisse Faculty, University of Bern, CH-3012 Bern, Switzerland; 6 School of Life Sciences, Ecole Polytechnique Fédérale de Lausanne, CH-1015 Lausanne, Switzerland

**Keywords:** Neurodegeneration, Alzheimer’s disease, APMAP-KO, Learning and memory, APMAP interactome, Aβ production, Alternative splicing

## Abstract

The adipocyte plasma membrane-associated protein APMAP is expressed in the brain where it associates with γ-secretase, a protease responsible for the generation of the amyloid-β peptides (Aβ) implicated in the pathogenesis of Alzheimer’s disease (AD). In this study, behavioral investigations revealed spatial learning and memory deficiencies in our newly generated mouse line lacking the protein APMAP. In a mouse model of AD, the constitutive deletion of APMAP worsened the spatial memory phenotype and led to increased Aβ production and deposition into senile plaques. To investigate at the molecular level the neurobiological functions of APMAP (memory and Aβ formation) and a possible link with the pathological hallmarks of AD (memory impairment and Aβ pathology), we next developed a procedure for the high-grade purification of cellular APMAP protein complexes. The biochemical characterization of these complexes revealed a series of new APMAP interactomers. Among these, the heat shock protein HSPA1A and the cation-dependent mannose-6-phosphate receptor (CD-M6PR) negatively regulated APP processing and Aβ production, while clusterin, calnexin, arginase-1, PTGFRN and the cation-independent mannose-6-phosphate receptor (CI-M6PR/IGF2R) positively regulated APP and Aβ production. Several of the newly identified APMAP interactomers contribute to the autophagy-lysosome system, further supporting an emergent agreement that this pathway can modulate APP metabolism and Aβ generation. Importantly, we have also demonstrated increased alternative splicing of APMAP and lowered levels of the Aβ controllers HSPA1A and CD-M6PR in human brains from neuropathologically verified AD cases.

**Electronic supplementary material:**

The online version of this article (10.1186/s40478-019-0660-3) contains supplementary material, which is available to authorized users.

## Introduction

Evidence gathered over the past thirty years has implicated the amyloid-β peptides (Aβ) as the causative agents in the pathogenesis of Alzheimer’s disease (AD) [9, 16]. Enhanced production associated with impaired clearance of Aβ and the consequent peptide polymerization into soluble oligomeric and/or insoluble amyloid deposits is indeed a critical and early event that triggers a succession of pathological reactions including hyperphosphorylation of tau and formation of neurofibrillary lesions, neuroinflammation, and neuronal death, ultimately leading to dementia [2, 23, 24, 31, 51]. Since Aβ peptides are derived from the proteolytic processing of the amyloid precursor protein (APP) by γ-secretase [20, 22], inhibiting the latter protease is a valuable approach that has been extensively tested in the clinic to prevent and/or delay the pathogenic effects of AD [38]. The significant adverse effects described in clinical studies [53, 54] have revealed the gaps and urgent needs in understanding the molecular and cellular pathways that regulate the activity of γ-secretase, APP processing and Aβ production in early- and late-onset AD in order to design safe and potent drugs against AD.

Previously, in a study that aimed to characterize the γ-secretase interactome, we have demonstrated that the adipocyte plasma membrane associated protein (APMAP, C20orf3), the expression of which is necessary for the maturation of adipocytes to acquire their capacity to store lipids [49], is also highly expressed in the brain, where it can physically interact with the γ-secretase complex and can function as a suppressor of Aβ production [40].

In this study, we first generated a constitutive knockout APMAP mouse line (APMAP-KO) that we characterized in a battery of morphologic and behavioral tests, to investigate the physiological role of APMAP *in vivo*. We next developed a procedure for the high-grade purification of cellular APMAP protein complexes and further assessed the ability of newly identified APMAP-interacting proteins (AIPs) to modulate APP processing and Aβ production. Finally, we investigated the physiological relevance of our findings in human brains from neuropathologically verified AD cases.

## Materials and methods

### Generation of the APMAP-KO and APMAP-KO/AD mouse lines

Embryonic stem cells (ESCs) carrying the APMAP exon 4 as described in the knockout-first construct (see Additional file 1 Figure S1) and with the C57Bl/6N genetic background were ordered from the Komp repository (Apmap^tm1a(KOMP)Wtsi^, KOMP repository, Davis, CA, USA). The transgene integration sites were confirmed by PCR and Southern blotting, and the ESCs were injected into C57Bl/6N blastocysts and implanted into pseudo-pregnant females. The chimera was bred for one generation with C57Bl/6N mice and further inbred to obtain the full constitutive knockout APMAP-KO mouse line and the control APMAP-WT line. The forward primer 5’-AGAGGAGCTTATGAGAGAGTTAATGG-3’ combined with the reverse primer 5’-TTGGTAAGAAAGGAAGCCAG-3’ were used for the detection of the wild type allele (530 bp insert), while the forward primer 5’-AGAGGAGCTTATGAGAGAGTTAATGG-3’ combined with the reverse primer 5’-CCAACTGACCTTGGGCAAGAACAT-3’ were used for the detection of the KO allele (726 bp insert). The APMAP-KO/AD mouse line was generated by breeding the APMAP-KO mice with the APPSwe-PS1dE9 mouse model for AD [30], then inbred for one generation to obtain a mouse line homozygous for APMAP-KO and heterozygous for the AD transgenes APPSwe-PS1dE9 (APMAP-KO/AD). Similarly, the control APMAP-WT line was bred with the AD mouse line to generate the control APMAP-WT/AD mouse line. Since the APMAP-KO and the APPSwe-PS1dE9 lines are of C57Bl/6J and C57Bl/6N genetic backgrounds, respectively, the APMAP-KO/AD line was maintained under a 1:1 mixed genetic background C57Bl/6N and C57Bl/6J. All mice were maintained at 23±1°C in a temperature-controlled facility, with a 12h light/dark cycle and were fed ad libitum. All animal experiments described in this study were approved by the veterinary ethics committee of the canton of Vaud - Switzerland (License IDs 2746).

### Pathophysiological characterization of the APMAP-KO mice

WT and APMAP-KO mice (chow or high fat diets, 5-9 months old) were euthanized by carbon dioxide inhalation, and further dissected. All organs listed in Additional file 1 Figure S2 were fixed for 48h in formalin (Sigma Aldrich, Buchs, Switzerland) and embedded in paraffin. Next, slices (4μm thickness) were prepared by using a cryostat (Leica, Muttenz, Switzerland), and subjected to Hematoxylin & Eosin staining. Mounted slices were analyzed in a blind fashion by two European board veterinary pathologists (A.P. and C.G.).

### Behavioral characterization of the APMAP-KO mice

Nine months old APMAP-KO mice underwent a battery of behavioral tests, in a sequence intended to prevent interferences between different tests. To avoid phenotypes specific to one estrous cycle phase, female mice of each experimental group were housed in several cages, thus avoiding estrous cycle synchronization. The *Morris water maze* test was performed to assess spatial learning and memory proficiency, as described previously [13, 59]. By using visual cues, mice had to learn the position of an escape platform (11 cm diameter) submerged 0.5 cm below the water surface and set in the center of the North quadrant of a circular pool (165 cm diameter). Water was kept at 24±1°C and made opaque by adding milk. The tank was placed in a room with artificial lighting set at 55 lux. Mice received 4 training trials a day during four days. Each trial started with a mouse released in the pool from a different point, alternating release points close and far from the escape platform. Mice not finding the platform within a delay of 120 s were gently accompanied to the platform and kept there for further 15 s. At the end of each trial, the mice were placed under a heating lamp for recovery in their home cages (inter-trial interval: 30 min). Retention of place learning was tested at day 5 with a 120 s probe trial where the escape platform was removed. Escape path lengths during training trials, and time spent searching in the four quadrants during the probe trial were assessed using a video tracking system (EthoVision 3.0, Noldus, Wageningen, NL). The *fear conditioning* test was performed to assess associative fear learning and memory, as previously described [46]. During the training session (1st day), the mice were placed in a conditioning chamber (Med Associates inc., Fairfax, VT, USA) where a tone (5000 Hz, 80dB) was presented for 30 s, and a foot-shock (0.7mAmp) delivered during the last 2 s of the tone presentation. Tone/foot-shock pairings were repeated four times. The second day, mice were placed in the same chamber and the freezing responses to the context, in absence of the tone/foot-shock, was monitored by using a video tracking system (EthoVision 3.0). The third day, the same mice were one more time exposed to the tone in a different context (Med Associates inc., Fairfax, VT, USA), and the freezing response to the auditory cue was assessed as described above.

Proficiency in incidental learning and short-term memory was assessed with *the novel object recognition* task [21]. Briefly, after familiarization to a white square arena (50 × 50 × 37 cm), mice were presented with two identical plastic objects to explore during 10 minutes. After 3h, mice were re-introduced in the same arena and presented with one identical and one novel object, placed in the same positions as in the previous presentation. Time spent in close proximity with the objects was assessed by a video tracking system. Novelty recognition results in increased contacts with the novel as compared to the familiar object. The *elevated plus maze* test was used to assess anxiety phenotypes, as described in previous studies [3, 17]. The maze was set-up 74 cm above the floor, with two open (stressful) and two enclosed (protecting) arms, designed in such a way that the arms of the same type are facing each other and remain connected by an opened central platform. In this test, security is provided to the mice by the closed arms (19 cm high side walls) whereas the open arms offer exploratory value. To prevent mice slipping off the maze, open arms have 0.5 cm high plexiglas rims. For the test, mice were placed in the central area and allowed to explore the maze for 10 min. A video tracking system (EthoVision 3.0) allowed the recording of the time spent in each arm. The *open-field* test was performed to assess exploration and locomotion, as described previously [56]. Briefly, mice were released in the center and left to freely explore a novel white square arena (50 × 50 × 37 cm), under dim light conditions (25 lux). Distance traveled, as well as time spent in the center (stressful) and peripheral (protecting) zones of the arena were recorded during 30 min by using a video tracking system (EthoVision 3.0).

### Place learning and spatial memory in APMAP-KO/AD mice

**Fig. 1:**
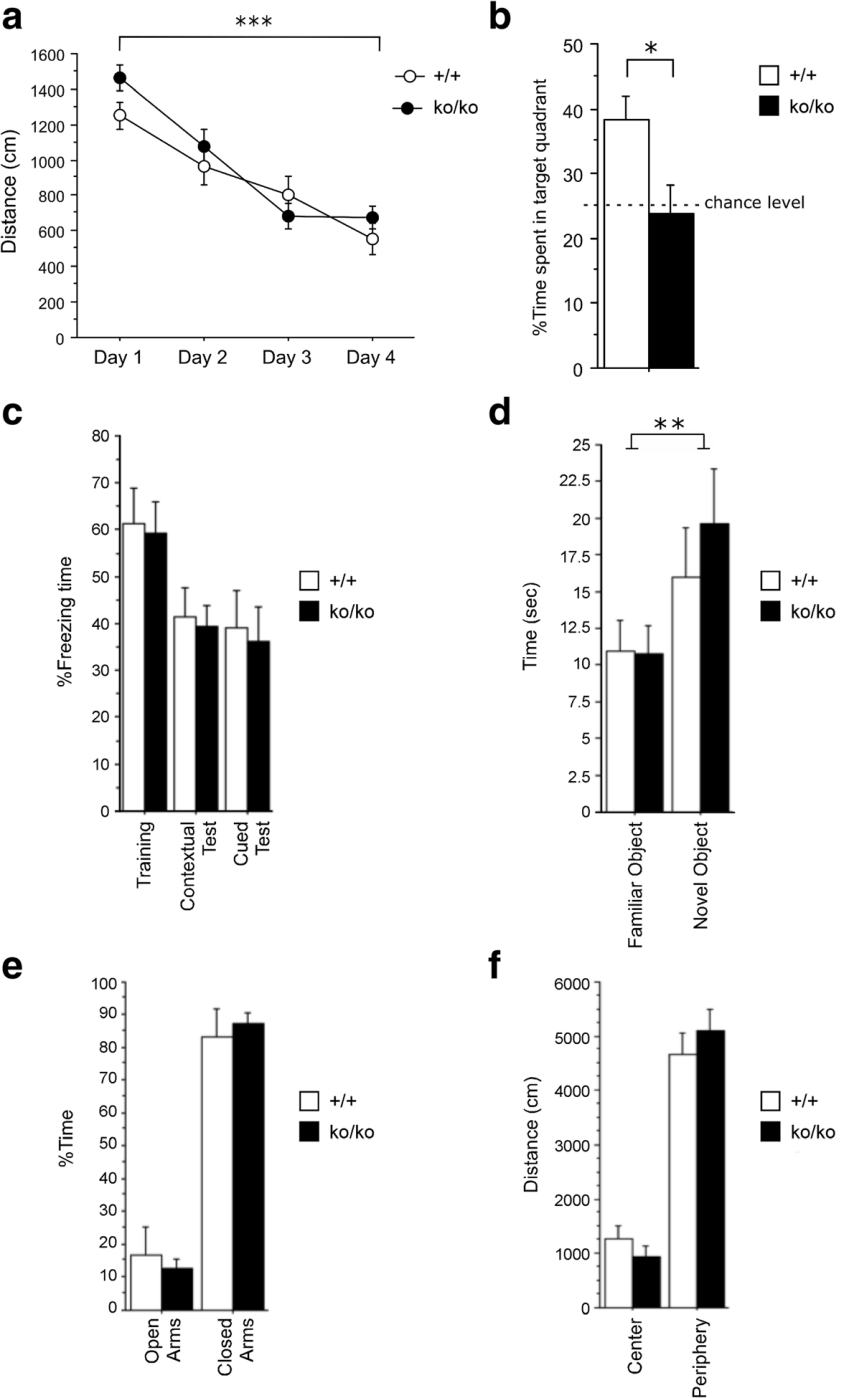
The constitutive deletion of APMAP selectively affects spatial memory but not anxiety, locomotion, fear-related or independent hippocampus memories in WT mice. **a**, **b** In the Morris water maze, 9-month-old WT (+/+; n=11; 6 females and 5 males) and APMAP-KO mice (ko/ko; n=13; 6 females and 7 males) show similar escape learning during training trials (repeated measures ANOVA: time effect F_3,22_=45,224, *** p<0.001 Day 1 versus Day 4) (**a**). However, in the spatial memory probe test, after 4 days of acquisition training, APMAP-KO mice performed at a chance level in the search for the platform, showing no preference for the target quadrant compared to WT control mice (**b**). **c** APMAP-KO mice did not demonstrate a deficit in the acquisition or retention of a fear-conditioning task (repeated measures ANOVA F_1, 22_=0.112, p>0.05 for genotype). **d** In an object recognition task, both groups spent more time exploring the novel object and were able to discriminate it from a familiar object (repeated measures ANOVA F_(1, 20)_=13.175, ** p<0.01 familiar vs novel object, Tukey post hoc test. Genotype effect F_(1, 20)_=0.660 p>0.05, interaction genotype x object F_(1, 20)_=1.211 p>0.05). **e** In the elevated plus maze, WT and APMAP-KO mice exhibited a similar exploration time on the open arms (repeated-measures ANOVA F_(1, 19)_=3.576, p>0.05 for genotype). **f** APMAP-KO mice did not exhibit a reduced locomotion activity or an anxiety-related behavior compared to WT mice during an open field test (repeated-measures ANOVA F_1,22_=0.7, p>0.05 for genotype). Data are expressed as the mean ± SEM

**Fig. 2:**
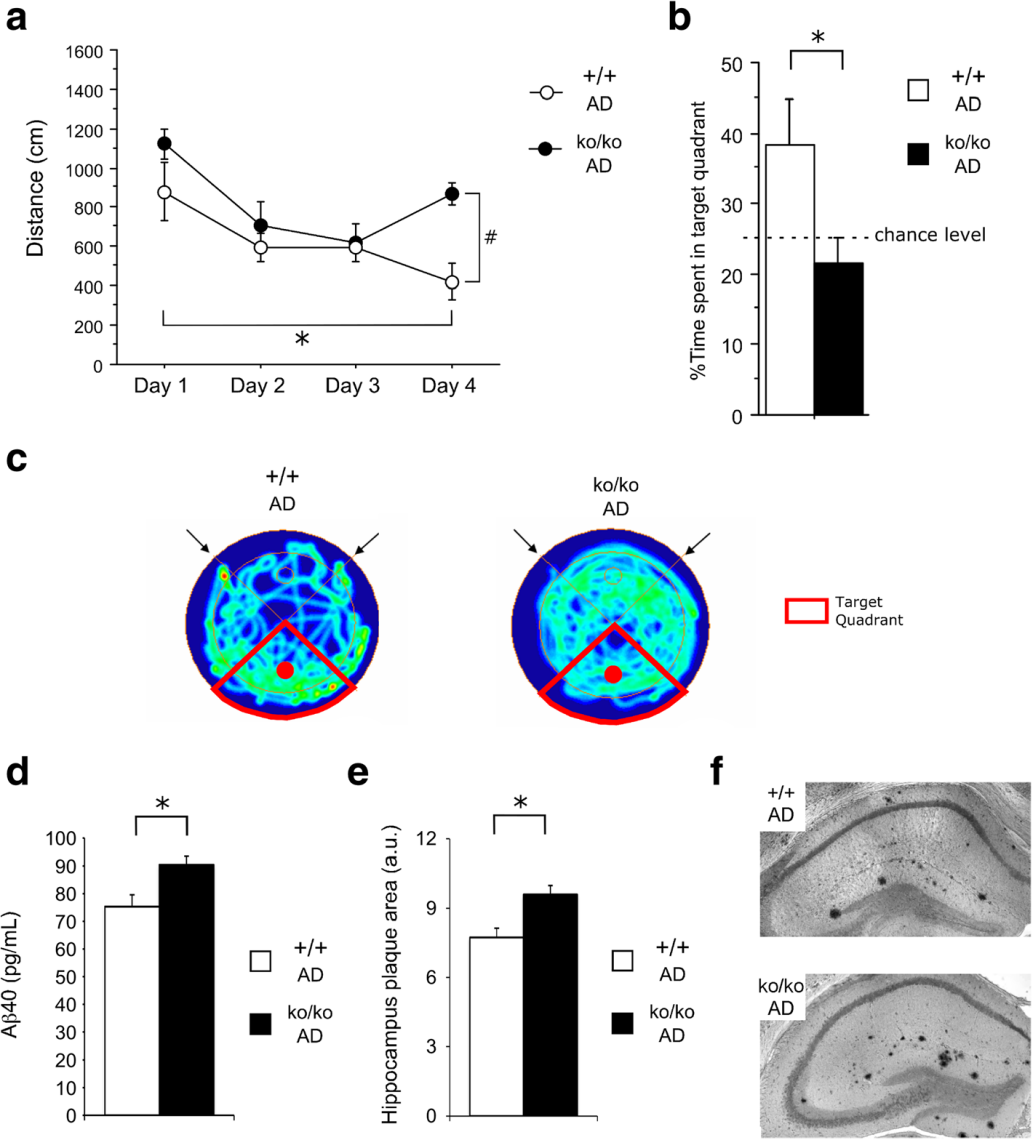
The constitutive deletion of APMAP worsens spatial memory and increases the hippocampal Aβ plaque load in AD mice. **a**, **b** In the Morris water maze, 20-month-old Alzheimer’s disease (AD) mice depleted for APMAP (ko/ko AD; n=5; 2 females and 3 males) exhibited poorer escape learning during training trials (repeated measures ANOVA: distance to platform, F_3,21_=8.426 *p<0.05 Day 1 versus Day 4; treatment effect F_1,21_=7.290, #p<0.05 +/+ AD versus ko/ko AD at day 4) (**a**), and spent significantly less time in the target quadrant during probe test (**b**) compared to AD control mice (+/+ AD; n=4; 1 female and 3 males). **c** Heat maps describing the spatial distribution of the two groups of animals during the probe trial. Arrows indicate release points; the solid circle indicates the platform position. **d** Aβ1-40 peptides were estimated by ELISA in whole brain SDS extracts prepared from the right hemispheres of 9-month-old APMAP-KO/AD mice (ko/ko AD; n=7 females) and age-matched wild-type control littermates (+/+ AD; n=4 females). **e** The detection of Aβ plaques was performed by immunohistochemistry (IHC) in the hippocampi of the left hemispheres of the same mice as in (**d**). **f** Representative microscopic images of coronal sections of hippocampi stained by IHC for the detection of Aβ deposits (in black). Student’s t-test was applied for statistical analyses in panels **d** and **e**, with * p<0.05

Cognitive proficiency was assessed in the APMAP-KO/AD mice at the age of 20 months, in the Morris water maze. Due to the expected lower proficiency of these mice, the protocol used for the APMAP-KO mice and described above was simplified as follows: mice were tested in a smaller pool (150 cm diameter) using a slightly larger platform (14 cm). In order to prevent fatigue, and improve learning, maximal escape latencies were shortened to 90 s, and mice underwent 6 instead of 4 learning trials per day. Consequently, the results in Fig. 1 and Fig. 2 shall not be compared directly. Path lengths to reach the platforms during training trials, and time spent in quadrants during the probe trial were assessed using a different video tracking system (AnyMaze, Ugo Basile, Varese, Italy).

### Immunohistochemistry and Aβ plaque quantification

Nine month-old APMAP-KO/AD and their control APMAP-WT/AD mice were euthanized and the brain’s left hemispheres were collected, fixed for 48h in 4% paraformaldehyde diluted in PBS (Roche), cryoprotected for 24h in 30% sucrose diluted in PBS, and kept at -80°C in the cryopreservative matrix. Coronal hippocampal slices of 20μm thickness were obtained with a cryostat, and further processed in a free floating manner. First, slices were washed in PBS, boiled for 20 min in citrate buffer (Invitrogen) and cooled at room temperature. Next, blocking, binding of the primary anti-Aβ 6E10 antibody (Biolegend, London, United Kingdom) and binding of the secondary anti-mouse HRP antibody, were performed with the Immpress kit (Vector laboratories, Burlingame, CA, USA) according to manufacturer instructions. Next, staining was performed by using the SG-blue chromogen kit (Vector laboratories), according to manufacturer instructions. Finally, individual Aβ plaques were scored on slices mounted with fluorsave (Millipore, Schaffhausen, Switzerland) and analyzed by phase contrast microscopy (Zeiss, Feldbach, Switzerland).

### Cell Lines and Cultures

Human embryonic kidney cells (HEK 293T) and human cervical carcinoma cells (HeLa) cells were routinely grown on plates in Dulbecco’s modified Eagle’s medium (DMEM) with 10% fetal bovine serum (FBS) and penicillin/streptomycin in a humidified 5% CO_2_ atmosphere. The CHO cells stably expressing Flag-tagged APMAP and the HEK cells stably expressing APP with the Swedish mutation (HEK-APPSwe [19]) were maintained in DMEM with 10% FBS supplemented with 150 μg/ml Geneticin G418. DMEM, FBS, Penicillin-Streptomycin and G418 sulfate were purchased from Invitrogen (Carlsbad, CA, USA). To adapt the CHO cells stably expressing Flag-tagged APMAP for small-scale (less than 50 ml) cultures in suspension, cells were inoculated at a density of 0.5 x 10^6^ cells/ml in 5 ml ProCHO5 pre-warmed medium (Lonza Verviers, Verviers, Belgium) containing 1% FBS in CultiFlask 50 tubes (Sartorius AG, Göttingen, Germany). The cultures were agitated for 3-4 days by orbital shaking at 180 rpm in an ISF-4-W incubator (Kühner AG, Birsfelden, Switzerland) at 37°C in the presence of 5% CO_2_ [41]. Large-scale 10-liter suspension cultures in ProCHO5 medium were performed in six 5-liter bottles (1.7 L per bottle) and agitated at 110 rpm at 37°C as above.

### Purification of APMAP and associated proteins from CHO-APMAP1-Flag cells

The multi-step procedure for the high-grade purification of native APMAP1 and associated proteins was performed as previously described for the purification of the γ-secretase complex [12]. Briefly, a total of 2.64 x 10^9^ CHO cells stably transfected with human APMAP1-Flag were resuspended in MES buffer (50 mM MES pH 6.0, 150 mM NaCl, 5 mM MgCl_2_, 5 mM CaCl_2_, and protease inhibitor cocktail (Roche)). The cells were lysed by three passages in a high-pressure homogenizer at a pressure greater than 1000 psi and centrifuged at 3000 x g for 20 min. The supernatant was further centrifuged at 100,000 x g for 1h to pellet membranes. The pellet was resuspended in bicarbonate buffer (0.1 M NaHCO_3_, pH 11.3) and incubated for 20 min at 4°C to remove non-integral proteins. The washed membranes were recovered by centrifugation at 100,000 x g for 1h. The supernatant was discarded, and the membranes were solubilized for 1h at 4°C in ice-cold lysis buffer (50 mM HEPES, 150 mM NaCl, 5 mM MgCl_2_, 5 mM CaCl_2_) containing 1% of 3-([3-Cholamidopropyl]dimethylammonio)-2-hydroxy-1-propanesulfonate (CHAPSO; Sigma-Aldrich, Steinheim, Germany), and a protease inhibitor cocktail (Roche, Basel, Switzerland). The lysate was centrifuged at 16,000 x g, saved, and diluted twice in HEPES buffer (50 mM HEPES, 150 mM NaCl, 5 mM MgCl_2_, 5 mM CaCl_2_, and protease inhibitor cocktail (Roche)). The lysate was further diluted six times in 0.1% digitonin-TBS buffer (50 mM Tris-HCl pH 7.4, 150 mM NaCl) and bound to M2 anti-Flag affinity resin (Sigma Aldrich) overnight. Following three washes in 0.1% digitonin-TBS buffer, the bound proteins were eluted in 1.5 ml of 0.1% digitonin-TBS buffer containing 0.2 mg/ml Flag peptides and finally subjected to size exclusion chromatography, as described below.

### Size exclusion Chromatography

The size exclusion chromatography of APMAP1 and associated proteins was performed on a Superdex 200 10/300 GL column (GE Healthcare, Wauwatosa, WI, USA), as previously described [40]. Briefly, the two APMAP fractions successively eluted from the M2 anti-Flag affinity resin (150 μl after 20-fold concentration) were loaded and eluted with 0.1% digitonin-TBS at 0.3 ml/min. The column was calibrated with soluble standards blue dextran 2000 (void volume), thyroglobulin (669 kDa) and ferritin (440 kDa), which were purchased from GE Healthcare (Wauwatosa, WI, USA).

### Tryptic digestion and mass spectrometry

The fractions collected after the size exclusion chromatography were run on a NativePAGE Novex® Bis-Tris 4-16% gel for BN-PAGE analysis (Invitrogen, Carlsbad, CA) and the APMAP-containing complexes were visualized by Silver staining, excised, and cut into small pieces. Proteins were reduced, alkylated, and subjected to in-gel digestion with trypsin. Briefly, gel pieces were destained, desiccated by incubating twice in 200 μl of 50 mM ammonium bicarbonate and 50% ethanol for 20 min, and dried with a vacuum concentrator. The samples were then incubated overnight at 37°C with trypsin (12.5 ng/μl). For liquid chromatography coupled to tandem mass spectrometry (LC-MS/MS) analysis after extraction from gel slices, peptides were resuspended in 2% Acetonitril / 0.1% Formic Acid and separated by reverse-phase chromatography on a Dionex Ultimate 3000 RSLC nanoUPLC system connected in-line with an Orbitrap Elite (Thermo Fischer Scientific, Waltham, MA). The instrument was operated in an information-dependent mode where peptide masses were selected for collision-induced dissociation (CID) to generate tandem mass spectra. A database search was performed using Mascot 2.3 (Matrix Science, Boston, MA) and SEQUEST in Proteome Discoverer v.1.3 against a human database (UniProt release 2013_01; 87613 sequences) and Mammalian database (UniProt release 2011_07; 91104 sequences). All searches were performed with trypsin cleavage specificity, with up to 3 missed cleavages allowed, an ion mass tolerance of 10 ppm for the precursor, and 0.5 Da for the fragments. Carbamidomethylation was set as a fixed modification, whereas oxidation (M), acetylation (protein N-term), and phosphorylation (STY) were considered variable modifications. Data were further processed and inspected using the proteome software Scaffold 3.

### siRNA treatments

For siRNA knockdown experiments, cells were treated for 3 days with the siRNAs listed in the table below. All siRNA duplexes were complexed with Lipofectamine RNAiMax from Invitrogen.

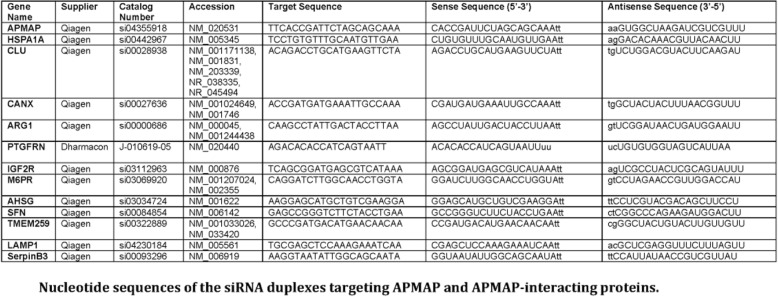



### Western blotting and antibodies

For Western blot analysis of mouse brain APMAP and associated proteins, brain total protein extracts were prepared in a 50 mM HEPES pH7.0 buffer (1mL/100mg tissue) containing 1% NP40 and a complete protease inhibitor cocktail (Roche). After tissue dissociation with a Bounce Homogenizer and centrifugation at 17’000g for 30 min at 4°C, the supernatant was collected and the equivalent of 40 μg of total proteins were loaded onto a 12% acrylamide Tris-Glycine gel and further resolved by SDS-PAGE and transferred onto nitrocellulose membranes. Next, the proteins APMAP and β-actin were detected by using anti-APMAP (4F6, AbCam, Cambridge, UK) and anti-β-actin (Sigma-Aldrich, Saint Louis, MS, USA) antibodies respectively. The secondary antibodies conjugated to Alexa 680 were purchased from Invitrogen, and the Odyssey infrared imaging system (LICOR, Lincoln, NE, USA) was used to detect the fluorescent signal. For Western blot analysis of cellular APMAP and associated proteins, whole cell extracts were prepared in 50 mM HEPES buffer containing 1% NP40 and complete protease inhibitor cocktail (Roche) and were run on 12% Tris-glycine PAGE gels, transferred onto PVDF membranes and probed with: antibody HPA012863 (for APMAP, 1:2000, Sigma-Aldrich, St. Louis, MO), A206 (for Actin, 1:2000, Sigma-Aldrich), CT15 (for APP-FL and APP-CTFs, 1:1000, Sigma-Aldrich), MAB1563 (for PS1-NTF, 1:1000, Chemicon International, Temecula, CA), Ab1997 (for ADAM10, 1:1000, Abcam, Cambridge, MA), EE-17 (for BACE1, 1:1000, Sigma-Aldrich, St. Louis, MO), AF5868 (for Arginase 1, 1:2000, R&D Systems, Minneapolis, MN), sc-6419 (for Clusterin alpha, 1:1000, Santa Cruz Biotechnology, Dallas, TX), AF1663 (for HSPA1A, 1:2000, R&D Systems), ADI-SPA-865-D (for Calnexin, 1:1000, Enzo, New York, NY), PA5-51134 (for PTGFRN, 1:1000, Thermo Fisher, Waltham, MA), AF5320 (for M6PR-CD, R&D Systems), AF2447 (for M6PR-CI, R&D Systems). Same approach was used for the Western blot analysis of APMAP and associated proteins in human frontal cortex tissue lysates.

### Aβ40 and Aβ42 quantitative assays by ELISA

Aβ1-40 and Aβ1-42 peptides secreted in the conditioned medium of HEK-APPSwe cells treated were quantitatively measured by ELISA, according to the protocol provided by the manufacturer (ELISA kits KHB3481 and KHB3441, Invitrogen).

### Human frontal cortex tissue lysates

Lysates from frontal cortex of frozen human brains from neuropathologically verified AD cases (obtained from the brain bank of the Alzheimer’s Disease Research Centre (ADRC) at Massachusetts General Hospital) were prepared by RIPA buffer with protease and phosphatase inhibitor cocktail (Fisher Scientific) and calyculin A (Cell Signaling Technology, Danvers, MA, USA), as described previously [37]. Control cases were non-demented individuals who did not meet pathological diagnostic criteria of AD or any other neurodegenerative diseases. More information that includes age, gender, post mortem interval (PMI), degree of pathology (Braak stages), standardized and validated clinical, neuropsychological, neuropathological and behavioral assessments of AD (CERAD) are provided in Table 1.

**Table 1:**
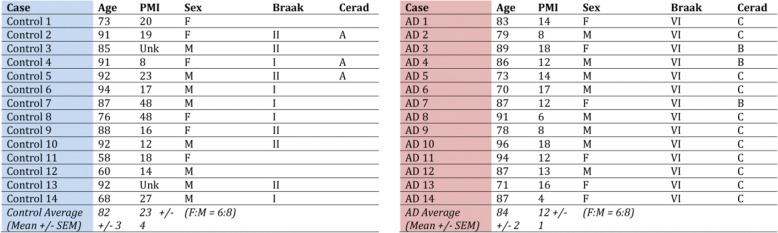
Demographic and diagnostic features of the human brain cortical samples used in this study

Age, gender, post mortem interval (PMI), degree of pathology (Braak stages) and standardized/validated clinical, neuropsychological, neuropathological and behavioral assessments of AD (CERAD) are provided for both control cases (left; non-demented individuals who did not meet pathological diagnostic criteria of AD or any other neurodegenerative diseases), or AD cases (right)

### Deglycosylation assays

RIPA-buffer was used for whole protein extraction of frozen human frontal cortical samples. The extracted proteins (1mg/mL) were then denatured by addition of SDS (Final conc. 1%) and heating at 75°C during 10 minutes. The denatured proteins were incubated with Peptide-N-glycosidase F (PNGase F; Sigma-Aldrich, P7367) during 1h at 37°C in order to remove N-inked oligosaccharides from glycoproteins.

### Label-free quantitative proteomics

The brain left hemispheres from 17 month old male APMAP-KO (n=3) or WT (n=3) mice were homogenized by using a Heidolph RZR2052 control device and total proteins were extracted from the tissue in RIPA buffer (150 mM NaCl, 1.0% NP-40, 0.5% sodium deoxycholate, 0.1% SDS, 50 mM Tris-HCl pH 8.0) and protease inhibitor cocktail (Roche). For each sample, 200 μg of solubilized proteins were reduced and alkylated before being loaded on 4-12% gradient gels (NuPAGE, Thermo Fisher) for protein separation. After staining (Colloidal Blue staining kit, Invitrogen LC6025), each gel lane was cut into 10 slices, the proteins were in-gel digested with trypsin (Promega) and the resulting peptide mixtures were processed on STAGE tips and analysed by LC-MS/MS [48, 52]. The LC-MS measurements were performed on a LTQ-Orbitrap mass spectrometer coupled to an EasyLC 1000 nanoflow-HPLC. Peptides were separated on fused silica HPLC-column tip (I.D. 75 μm, New Objective, self-packed with ReproSil-Pur 120 C18-AQ, 1.9 μm to a length of 20 cm) using a gradient of A (0.1% formic acid in water) and B (0.1% formic acid in 80% acetonitrile in water). The mass spectrometer was operated in the data-dependent mode; after each MS scan (mass range m/z = 375 – 1750; resolution: 60000) a maximum of five MS/MS scans were performed using normalized collision energy of 35% and a target value of 1000. The MS raw files were analysed using MaxQuant Software version 1.4.1.2 [4] for peak detection, quantification and peptide identification using a full length UniProt mouse database (April, 2016). Keratins and trypsin were used as references. Carbamidomethylcysteine was set as fixed modification and protein amino-terminal acetylation, lysine acetylation and oxidation of methionine were set as variable modifications. The MS/MS tolerance was set to 20 ppm and three missed cleavages were allowed using trypsin/P as enzyme specificity. Peptide and protein false discovery rate (FDR) based on a forward and reverse database were set to 0.01, minimum peptide length was set to 7 amino-acids, and minimum number of unique peptides for identification of proteins was set to one. The “match-between-run” option was used with a time window of 1 min. For normalization the MaxLFQ algorithm, part of the MaxQuant suite was used. Statistical analysis of all proteins revealed 113 significantly altered proteins (T-test, p-value  <  0.05; all information provided in Additional file 2 Table S1). In order to identify significantly enriched GO terms (p-value  <  0.05), the list of significantly altered proteins was analyzed against the whole list of detected proteins with the 1D enrichment tool in Perseus [58].

### Statistical analyses

For the behavioral analyses in Figs. 1 and 2, the repeated measures ANOVA test was applied for the statistical analyses, and the statistical significance is shown as *P*  <  0.05 (one asterisks), *P* < 0.01 (two asterisks) or *P* < 0.001 (three asterisks). For the other figures, the unpaired student’s *t*-test (two-tailed) was applied. All data are presented as mean ± SEM.

## Results

### APMAP-KO mice display spatial learning and memory deficiencies

We first generated an APMAP-KO mouse line with the knockout-first construct (Additional file 1 Figure S1) and completed a detailed pathological inspection on 4–9-month-old mice, which did not reveal any macroscopic and/or microscopic morphological abnormalities in peripheral and brain tissues (Additional file 1 Figure S2). Since APMAP is strongly expressed in the central nervous system [28, 40], we next conducted several behavioral tests to assess whether the deletion of this gene causes behavioral alterations. Spatial learning and memory was assessed in 9-month-old WT and APMAP-KO mice, in the Morris water maze performed as described in the Materials and Methods section. Mice from both groups showed comparable proficiency in water escape learning, significantly reducing their swim paths to the platform over the four training days (Fig. 1a). However, in the probe trial without a platform, performed 24 h after the last training session, only WT mice showed spatial searching in the target quadrant, while APMAP-KO mice performed at the chance level (Fig. 1b). Together, these results demonstrate spatial learning and memory deficiencies in APMAP-KO animals. Other forms of learning, such as Pavlovian conditioning and semantic memory, respectively assessed in cued and contextual fear conditioning and in an object recognition task, were not affected in the APMAP-KO mice (Fig. 1c, and d). Moreover, APMAP deletion did not affect anxiety, mobility, or exploratory drive, as estimated in the elevated plus maze and open field tests (Figs. 1e, and f).

### The constitutive deletion of APMAP worsens spatial memory and Aβ plaque deposition in a mouse model of AD

We next generated an AD mouse model lacking the APMAP gene (APMAP-KO/AD) by cross-breeding the APMAP-KO mice with APP/PS1 mice coexpressing the KM670/671NL Swedish mutation of human amyloid precursor protein (APP) and the dE9 mutation of human presenilin 1 (PS1), which develops parenchymal Aβ plaques starting at the age of 6 months [30, 35]. In the Morris water maze performed under simplified conditions when compared to WT mice (smaller pool and shorter trials - see Materials and Methods), the APMAP-KO/AD mice were less proficient than their control AD mice, with significantly longer swim paths on day 4 of the training (Fig. 2a), as well as showing a significantly lower target quadrant preference in the probe trial (Fig. 2b). Indeed, while WT/AD mice were sufficiently oriented on the target quadrant, APMAP-KO/AD mice did not show any spatial searching during the probe trial, demonstrating a more severe spatial memory deficit (Fig. 2c). Furthermore, extensive biochemical and immuno-histological analyses revealed a 20±4 % increase of cerebral Aβ1-40 levels (Fig. 2d) associated with a 24±5 % increase of the hippocampal Aβ plaque area in the AD mice lacking APMAP as compared to the control AD mice (Figs. 2e, and f). Altogether, our findings revealed subtle but important roles of APMAP in the learning and memory processes and in the production of Aβ peptides and their deposition into senile plaques.

### Purification and identification of APMAP interacting proteins

**Fig. 3:**
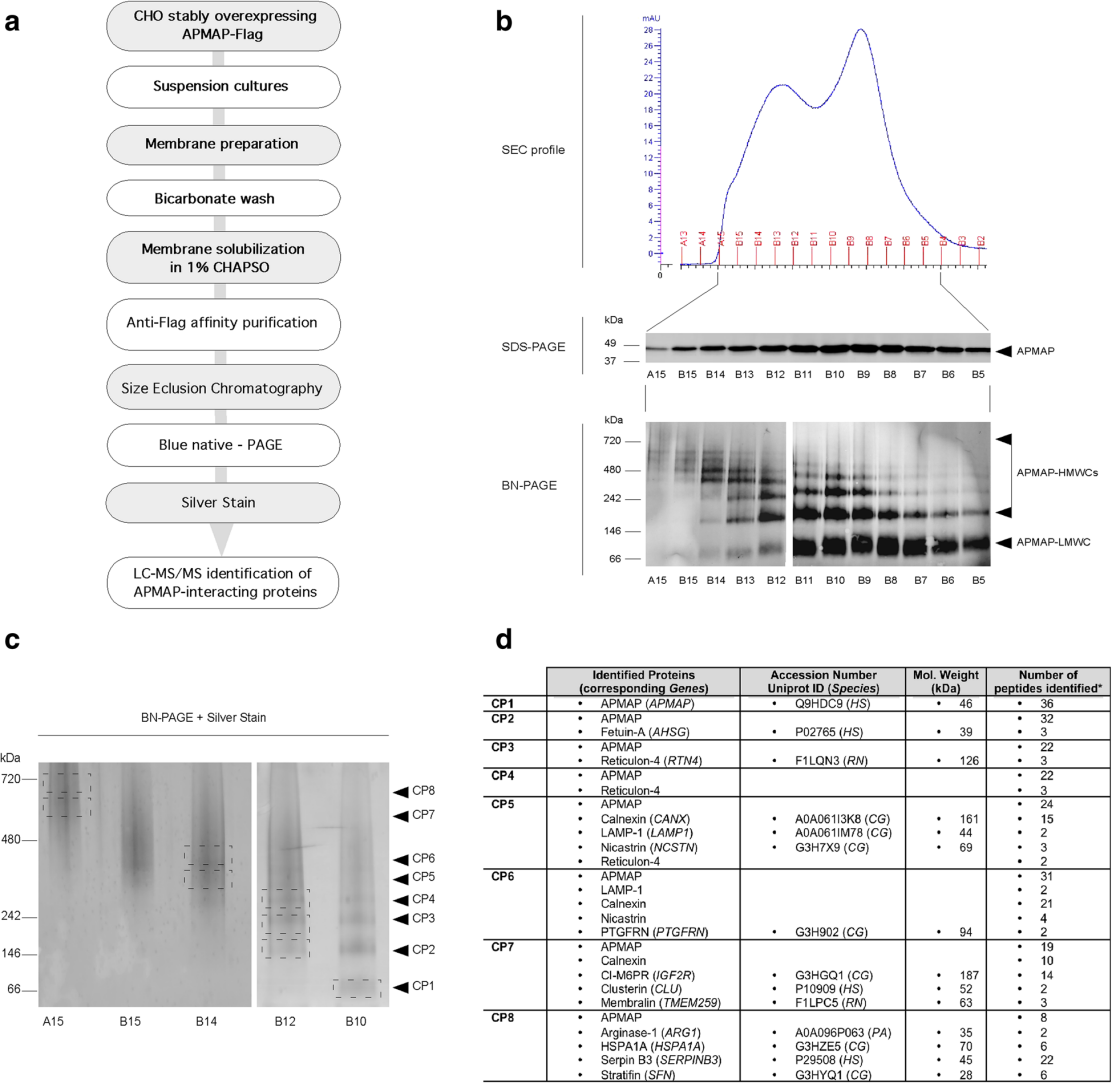
Purification of APMAP protein complexes and identification of APMAP interacting proteins. **a** Schematic representation of the multistep process designed for the purification of APMAP protein complexes and for the identification of APMAP interactomers. **b** Affinity purified APMAP protein complexes of different sizes were separated by size exclusion chromatography (SEC) on a Superdex 200 10/300 GL column. The SEC protein elution profile (*top*) revealed a protein distribution over 12 SEC fractions (A15 to B5), separated under denaturing conditions (SDS-PAGE) and immunostained with an anti-APMAP antibody (*middle*) or separated by blue native-PAGE (BN-PAGE) on a 4-16% Bis-Tris gel, and immunostained for APMAP (*bottom*). APMAP protein complexes appeared on the native gel as a low-molecular-weight complex (LMWC) of ~60 kDa and high-molecular-weight complexes (HMWCs) of ~150 to ~650 kDa. **c** Mass spectrometric identification of APMAP interacting proteins. The APMAP-containing low- and high-molecular-weight complexes from selected SEC fractions A15, B15, B14, B12 and B10 were resolved by native-PAGE on a 4-16% Bis-Tris gel, stained by silver nitrate, and the bands corresponding to eight different protein complexes (CP1 to CP8) were excised for protein content analysis by LC-MS/MS mass spectrometry. **d** Summary table of APMAP-interacting proteins identified by LC-MS/MS in CP1 to CP8. *Proteins and peptides identified by LC-MS/MS are listed in Additional file 1 Figure S4

We next designed a multistep purification procedure for the high-grade purification of APMAP and APMAP protein complexes (Fig. 3a). First, we generated, selected and adapted for cultures in suspension a CHO cell line that stably overexpressed APMAP1-Flag (Additional file 1 Figure S3). Then, large amounts of CHO-APMAP1-Flag cells harvested from a 10 L suspension culture were homogenized in a French press and cellular membranes were prepared by differential centrifugation and washing with a bicarbonate buffer [10] to remove both residual cytosolic proteins and peripheral membrane proteins [14], leaving only integral or tightly associated membrane proteins. The washed membranes were then solubilized in a buffer containing 1% CHAPSO, and the Flag-tagged APMAP and APMAP protein complexes were affinity purified following a previously described M2 anti-Flag antibody immuno-affinity procedure [40]. As a last purification step, the proteins eluted from the M2 resin were separated by size exclusion chromatography (SEC) and the fractions enriched in APMAP (Fig. 3b) were further loaded onto a Blue Native gel (BN-PAGE), which allows the detection of APMAP protein complexes in their native conformations. As shown in Fig. 3b, the BN-PAGE analysis of the SEC fractions revealed APMAP-containing protein complexes with apparent molecular masses ranging between ~60 kDa and ~650 kDa.

To identify proteins physically bound to APMAP, the SEC fractions enriched in APMAP protein complexes of different sizes (SEC fractions A15, B15, B14, B12 and B10 – see Fig. 3b) were resolved by preparative BN-PAGE and stained with silver nitrate (Fig. 3c). The bands corresponding to well-separated APMAP complexes (named CP1 to CP8) were excised for protein content analysis by LC-MS/MS mass spectrometry. A total of 13 APMAP-interacting proteins were identified in two independent experiments: fetuin-A, reticulon-4, calnexin, lysosome-associated membrane glycoprotein 1 (LAMP-1), nicastrin, prostaglandin F2 receptor negative regulator (PTGFRN), cation-independent mannose 6-phosphate receptor (CI-M6PR/IGF2R), clusterin, membralin, arginase-1, heat shock-related 70 kDa protein 2 (HSPA1A), serpinB3 and 14-3-3 protein sigma (stratifin) (Fig. 3d; protein sequences and peptides identified by LC-MS/MS are listed in Additional file 1 Figure S4).

### APMAP-interacting proteins modulate APP processing and Aβ production

The identification of nicastrin and reticulon-4 in the purified APMAP complexes confirms our previous observation that the proteins APMAP and reticulon-4 can physically interact with the γ-secretase complex [40]. Since nicastrin is the subunit of the γ-secretase complex that is responsible for substrate recognition [11], its genetic cellular depletion by small interfering RNA (siRNA) impairs both APP processing and Aβ production [8, 34]. By using a similar approach, we have previously shown that a drastic depletion of reticulon-4 was associated with increased APP-C-terminal fragments (APP-CTFs) and Aβ1-40 levels [40]. Based on these observations, nicastrin and reticulon-4 were omitted from further analysis in this new study.

**Fig. 4:**
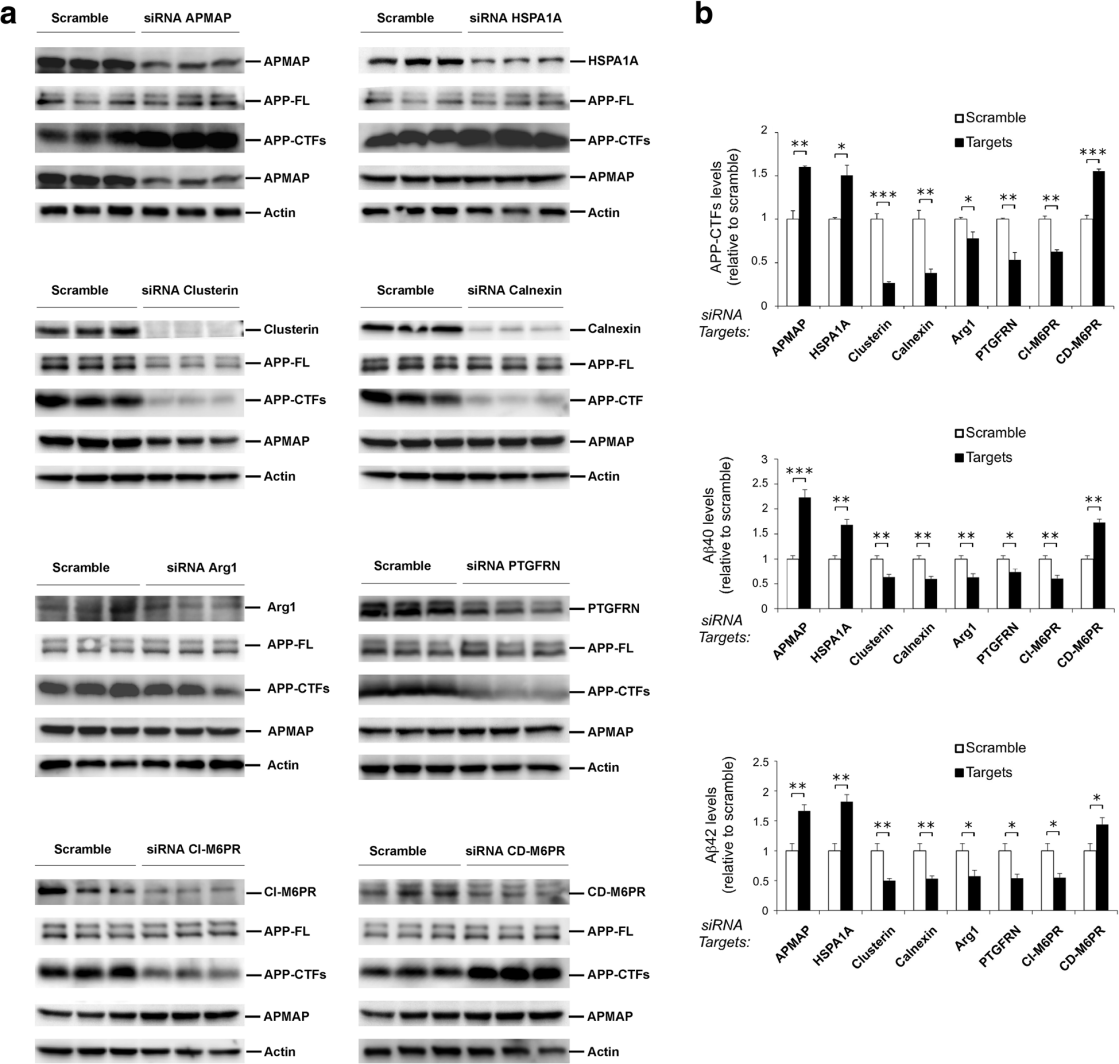
APMAP interacting proteins are endogenous modulators of APP processing and Aβ production. **a** The knockdown of the indicated APMAP-interacting proteins was mediated by siRNA in HEK cells overexpressing APP bearing the Swedish mutation that causes early-onset familial Alzheimer’s disease (HEK-APPSwe). After 3 days of treatment, whole cell extracts from biological triplicates were prepared and analyzed by Western blot for the siRNA protein targets, APP-FL, APP-CTFs and APMAP. Actin served as a protein loading control. Scramble: allstar control siRNA; APP-FL: APP full-length; APP-CTFs: APP-C-terminal fragments. siRNA duplexes are listed in the Materials and Methods section. **b** The conditioned media of the siRNA-treated cells in (**a**) were used to quantitatively measure, by ELISA, the secreted peptides Aβ1-40 and Aβ1-42. Note the correlation between APP-CTFs levels estimated by densitometric analysis of the APP-CTFs Western blot bands in (**a**) and the production of both Aβ1-40 and Aβ1-42. Student’s *t*-test was applied for statistical analysis; the significance is shown as the mean ± SEM, *P < 0.05; **P < 0.01; ***P < 0.001; Aβ40 and Aβ42: *n* = 6/group; APP-CTFs: n=3/group

To investigate whether the other newly identified APMAP-interacting proteins can also modulate the processing of APP and the production of the Aβ peptides, their expression was reduced by siRNA in APP-overexpressing HEK cells. Treatments with siRNAs targeting fetuin-A, stratifin, membralin, LAMP-1 and serpinB3 did not impact the levels or maturation of full-length APP (APP-FL), APP-CTFs, or APMAP (Additional file 1 Figure S5). In contrast, siRNAs targeting the heat shock protein HSPA1A and the cation-dependent mannose-6-phosphate receptor (CD-M6PR; functional homolog of CI-M6PR/IGF2R) caused a strong accumulation of APP-CTFs, while siRNAs against clusterin, calnexin, arginase-1, PTGFRN and CI-M6PR/IGF2R lowered APP-CTFs levels (Fig. 4a). Notably, siRNAs targeting clusterin lowered the levels of both APP-FL and APMAP (Fig. 4a) and none of the other siRNA knockdowns of individual genes interfered with the protein levels of α-, β- or γ-secretases, with the exception of clusterin and CD-M6PR, which respectively reduced ADAM10 levels and increased BACE1 levels (Additional file 1 Figure S6).

Next, the production of Aβ peptides was investigated by quantitatively measuring both Aβ1-40 and Aβ1-42 peptides secreted in the conditioned medium of the siRNA-treated cells. We found a strong correlation between the effects observed on intracellular APP-CTFs and the production of both Aβ1-40 and Aβ1-42 (Fig. 4b). Indeed, increased APP-CTFs observed with siRNAs targeting HSPA1A and CD-M6PR were associated with increased Aβ40 and Aβ42 levels, while reduced APP-CTFs observed with siRNAs targeting clusterin, calnexin, arginase-1, PTGFRN and CI-M6PR/IGF2R were associated with reduced Aβ40 and Aβ42 levels (Fig. 4b).

### Alterations of APMAP interacting proteins in AD human brains

**Fig. 5:**
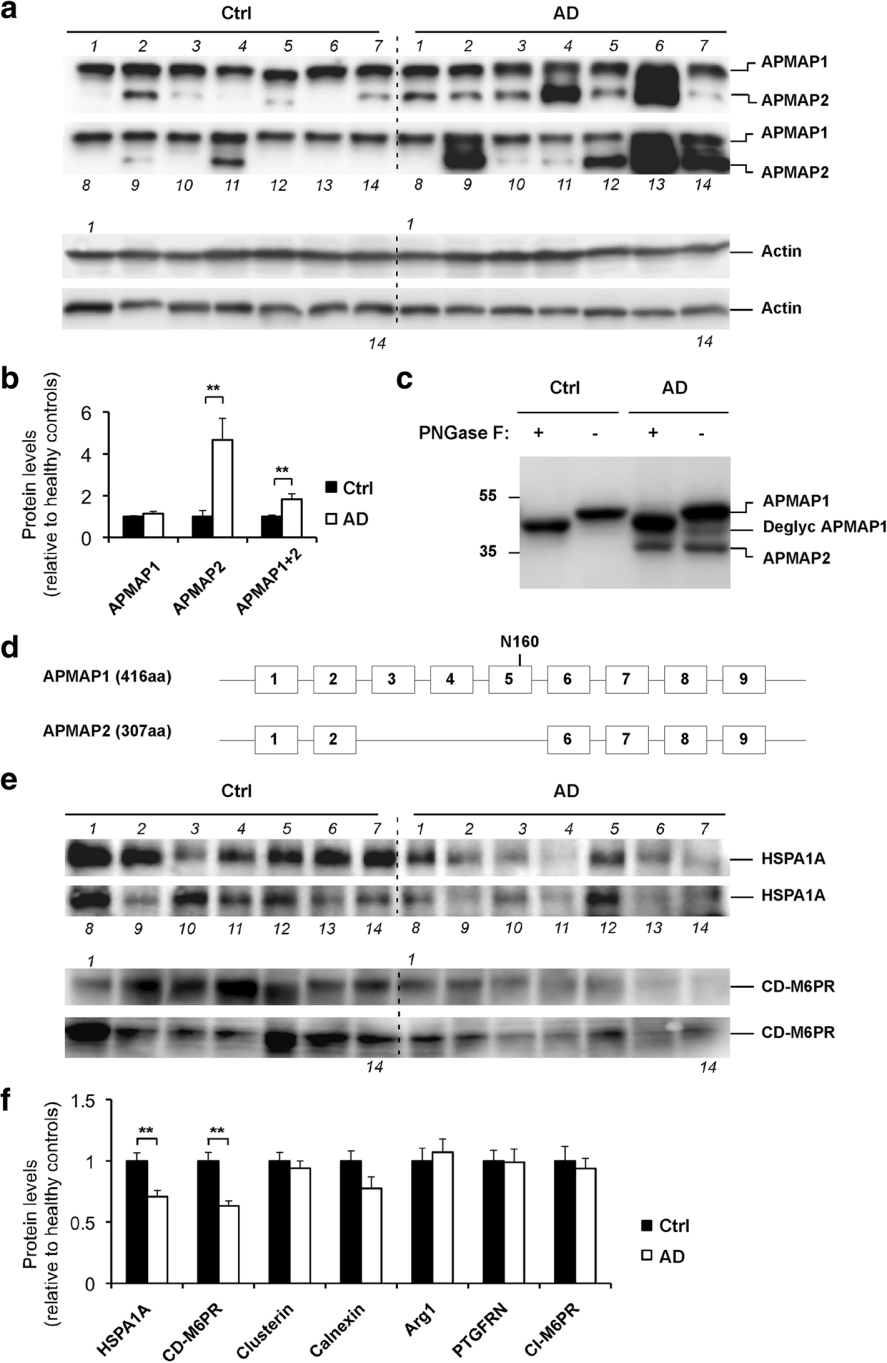
Increased alternative splicing of APMAP and reduced HSPA1A and CD-M6PR in AD brains. **a** Increased alternative splicing variant APMAP2 in AD brains, as estimated by Western blot analysis of APMAP1 and APMAP2 in cortical lysates of 14 control brains and 14 neuropathologically verified AD brains. Detailed demographic and diagnostic features of the human brain samples are provided in Table 1. Actin served as a loading control. **b** Densitometric analysis of the APMAP1 and APMAP2 Western blot bands in (**a**). Student’s t-test with mean ± SEM, **P < 0.01. **c** Denatured cortical lysates of control and AD brains treated in the presence (+) or absence (-) of PNGase. **d** Schematic representation of the exons and introns of APMAP1 and APMAP2. The predicted glycosylation site in exon 5 at position N160 is shown. **e** Reduced HSPA1A and CD-M6PR levels in AD brains, as estimated by Western blot analysis in the same samples as in (**a**). **f** Densitometric analysis of HSPA1A and CD-M6PR (**e**) and other APMAP-interactomers Additional file 1 Figure S9 Western blot bands. Student’s t-test with mean ± SEM, **P < 0.01

We next compared the protein profiles of APMAP and its newly identified interactomers in lysates of human brain cortical samples from neuropathologically verified AD cases and age-matched non-AD controls (Table 1). First, we found in the AD samples drastically enhanced levels (by 367±102%) of APMAP2 (Figs. 5a, and b), a previously reported alternative splice variant of APMAP1 that lacks exons 3, 4 and 5 [28]. PNGaseF treatment of the human lysates confirmed the glycosylation of APMAP1 and the absence of glycosylation in APMAP2 (Fig. 5c and Additional file 1 Figure S7), consistent with the unique APMAP1 glycosylation site predicted at position N160 (Additional file 1 Figure S8) in exon 5 that is missing in APMAP2 (Fig. 5d). Importantly, we also found in the AD samples significantly reduced levels of HSPA1A and CD-M6PR (the two negative regulators of Aβ production), by 29±5% and 37±4%, respectively (Figs. 5e, and f). No significant changes were found for the other APMAP-interacting proteins (Additional file 1 Figure S9).

### Brain proteome changes in APMAP-KO mice

**Fig. 6:**
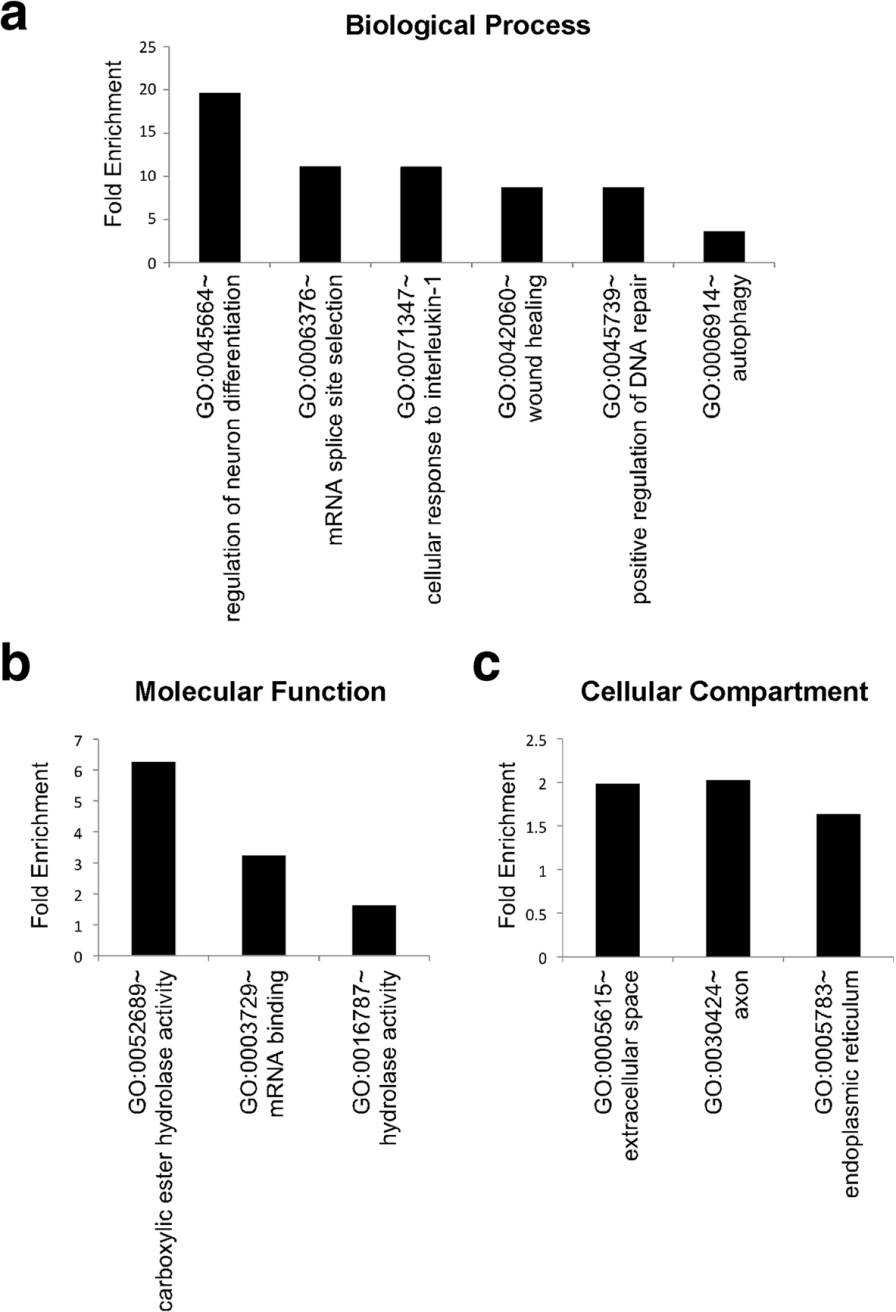
Representation of gene ontology enrichment in mice with constitutive depletion of APMAP, according to biological process (**a**), molecular function (**b**) and cellular compartment (**c**). Label-free quantitative proteomics (procedure described in details in the Materials and Methods section) was used to identify proteins differentially expressed in the brains of APMAP-KO mice. Significantly enriched GO terms (*p*-value < 0.05) were identified by comparing the list of 113 significantly altered proteins (Additional file 2: Table S1) against the whole list of 2747 detected proteins (Additional file 2: Table S1) using the 1D enrichment tool in Perseus

Finally, we applied label-free quantitative mass spectrometry to profile the brain proteome of APMAP-KO mice. This approach revealed 113 proteins differentially expressed in the brains of APMAP-KO mice (listed in Additional file 2 Table S1), thus suggesting novel neurobiological functions for APMAP and the APMAP interactome, including the regulation of neuronal differentiation, mRNA splicing, and autophagy (Fig. 6).

## Discussion

In this study, we show that the constitutive knockout of APMAP affects the hippocampal-dependent episodic memory, while other cognitive competences (procedural learning, semantic and pavlovian associations) are spared (Fig. 1). We also found that the deletion of APMAP in an AD mouse model results in a worsening of the spatial learning, and this in spite of a simplified water maze procedure that allowed sufficient place learning for the control AD mice (Fig. 2). We also demonstrate that the lack of APMAP increased the production of Aβ peptides and their aggregation into senile plaques in the hippocampus of an AD mouse model (Fig. 2). In order to investigate the molecular bases for these observations, we decided to develop a procedure for the characterization of the APMAP interactome. We found that cellular APMAP is organized into protein complexes of different sizes and we identified new APMAP interactomers, some of which modulate APP processing and the production of Aβ peptides (Figs. 3 and 4).

Amongst these, we identified nicastrin, a subunit of the γ-secretase complex [11], further supporting our previous observation that APMAP and the γ-secretase complex can associate into a HMW protein complex [40]. In addition, we identified reticulon-4 in APMAP complexes. Reticulon-4 is a myelin-associated membrane protein (also known as “Nogo”) that inhibits neurite outgrowth and limits plasticity in the healthy adult brain and neuronal regeneration during brain injury (for a review, see [57]). The co-purification of reticulon-4 with γ-secretase [40], with the β-secretase BACE1 [42] and with APMAP (this study) suggests potential microdomains (likely in the trans-Golgi network) made of the secretases, APP-FL and -CTFs, APMAP and reticulon-4.

Amongst the newly identified APMAP interactomers, HSPA1A and CD-M6PR, together with APMAP, were found to negatively regulate APP processing and Aβ production (Fig. 4). In contrast, clusterin, calnexin, arginase-1, PTGFRN and CI-M6PR positively regulated APP processing and Aβ production (Fig. 4). HSPA1A is a major protein of the Hsp70 family made of molecular chaperones that are critical for the cellular management of environmental stresses by preventing or reversing abnormal protein folding or aggregation (for reviews see [26, 29, 50]). Interestingly, recent studies have reported that dysfunctions or inhibitions of HSPA1A cause neurodegeneration, mainly by affecting the structure and function of the endosomes/lysosomes (for reviews, see [45, 62]). These observations are of particular interest since we found that reduced HSPA1A leads to increased production of APP-CTFs and Aβ peptides (this study - see Fig. 4), and since APMAP can regulate APP processing and Aβ production through the lysosomal-autophagic system [40]. Altogether, it is tempting to hypothesize that the physical and/or functional association between HSPA1A and APMAP may control APP processing and Aβ production by modulating the lysosomal activity through some yet unidentified molecular mechanisms.

The Prostaglandin F2 receptor negative regulator PTGFRN (also known as CD9P-1) is a member of the tetraspanin web that plays different biological functions including cell migration and cell fusion (for a review see [27]). Moreover, PTGFRN was recently identified in purified γ-secretase preparations [40, 60] while its receptor CD9 also co-immunoprecipitated with the active γ-secretase complex [60]. In the present study, we demonstrate for the first time that PTGFRN is part of the APMAP interactome, and propose that this association explains the functional role of PTGFRN in the regulation of APP processing and Aβ production (Fig. 4).

Clusterin is a major inflammatory-related apolipoprotein (Apolipoprotein J; ApoJ) that plays a protective role against apoptosis, cell damage, or oxidative stress [61]. Notably, recent genome-wide association studies from different groups have further uncovered clusterin variants that strongly associate with late-onset AD [25, 33]. Intriguingly, we found that reduced clusterin triggered reduced APMAP protein levels (Fig. 4), suggesting for the first time the existence of a common mechanism co-stabilizing these proteins. Moreover, clusterin has previously been reported to physically associate with the paraoxonase PON1 [1, 32, 36] whereas the only proteins sharing high sequence and structural homologies with APMAP are the three members of the PON family [28]. Together, these observations suggest a possible physical association between APMAP and clusterin, that regulates Aβ production/secretion through a molecular mechanism that needs further investigation, and that potentially involves the recently discovered function for clusterin in the biogenesis and activation of the autophagy-lysosomal system [63].

Importantly, we further observed that the reduction of CI-M6PR/IGF2R expression lowered APP-CTF/Aβ, whereas reduction of CD-M6PR expression (a functional homolog of CI-M6PR/IGF2R) caused a strong accumulation of APP-CTF/Aβ. Although the two M6PRs share common tasks that are essential for normal cellular function, including the delivery from the trans-Golgi network to the lysosomes of newly synthesized acid hydrolases [15], it is important to notice that the opposite results that we observed for APP-CTF/Aβ (Fig. 4) provides evidence that the receptors can fulfill different functions. Differences in the structural organizations of both M6P receptors (reviewed in [15]) may for example trigger the formation of separate transport vesicles having their own functional properties.

Altogether, these observations support the notion that several members of the APMAP interactome, including APMAP, HSPA1A, CD-M6PR and clusterin, may prevent Aβ production by developing interconnected functions that promote autophagy/lysosomal activity and facilitate the autophagy/lysosomal transport and degradation of the Aβ precursor protein substrates APP-CTFs. Further supporting this notion, two studies from different groups have recently revealed physical interactions between APMAP and APP [55] as well as between APMAP and the APP binding protein FE65 [43]. Even more recently, the role of alternative splicing in aging has emerged (for a review, see [7]) and alternative splicing events associated with AD have recently been reported for genes in the autophagy-lysosomal pathway [47]. In support to these observations, we found that the alternative splicing process of APMAP was increased in the brains of neuropathologically verified AD cases (Fig. 5).

Finally, a mass spectrometric-based quantitative analysis of the APMAP-KO brain proteome revealed more neurobiological functions for the APMAP interactome that include the regulation of neuronal differentiation, mRNA splicing, and autophagy (Fig. 6). Because neuronal differentiation is required for memory formation [6, 18], the alteration of this function (Fig. 6) provides a plausible explanation for the reduced learning and spatial memory phenotypes observed in APMAP-KO mice (Figs. 1 and 2). In addition, the possible implication of APMAP in neuronal differentiation (Fig. 6) together with the known function of peripheral APMAP in the differentiation of adipocytes [49], suggest a more general role for APMAP in cellular differentiation. At the molecular level, it was previously shown that the γ-secretase-dependent processing of APP and several other known substrates including the Notch-1 receptor or Neurexins are critical events for neuronal differentiation [5, 39, 44]. Thus, the possibility exists that the APMAP interactome may regulate neuronal differentiation through the modulation of the processing, by γ-secretase, of many different substrates. Further investigation is needed to verify this hypothesis, with a first emphasis on the processing of the Notch and Neurexin receptors.

Related to the above-described function for APMAP1 in protein trafficking, one can hypothesize that the increased APMAP2 expression and altered APMAP interactome observed in AD patients (Fig. 5) may potentially be involved in APP/APP-CTFs mis-trafficking and overproduction of Aβ. Based on these observations, further investigation is required to elucidate the potential role of APMAP2 in Aβ production and in the etiology of AD. More specifically, the role of APMAP2 in the regulation of APP-CTFs/Aβ could be investigated through overexpression in cellular models of APMAP2 and simultaneously depletion of CD-M6PR and HSPA1A, to recapitulate the phenotype observed in human AD brain samples (Fig. 5). Moreover, a similar biochemical approach as that used for the elucidation of the APMAP1 interactome (Fig. 3) could be used for the comparison of the isoform-specific APMAP1 and APMAP2 interactomes. Next, the influence of the two splicing variants APMAP1 and APMAP2 on the trafficking and processing of APP and other γ-secretase substrates could be assessed. Finally, a correlation study between the APMAP2 expression and Aβ pathology, with a larger panel of human samples, would make a stronger case for the importance of APMAP in AD pathology.

Overall, the characterization in this study of the APMAP interactome and the identification of new endogenous modulators of Aβ production not only offer new therapeutic targets for the development of new treatments for neurodegenerative or memory disorders, but they also help to better understand the pathobiological processes of the sporadic, age-related forms of AD.

## Additional files

Additional file 1: **Figure S1.** Generation of the APMAP-KO mouse line. **Figure S2.** Morpho-pathological characterization of APMAP-KO mice. **Figure S3.** Generation and selection of a CHO cell line stably overexpressing APMAP for the high-grade purification of APMAP and associated proteins. **Figure S4.** Mass spectrometric identification of APMAP-interacting proteins. **Figure S5.** Treatments of HEK-APPSwe cells with siRNAs targeting fetuin-A, serpinB3, stratifin, LAMP-1 and membralin do not affect APP-FL, APP-CTFs, or APMAP. **Figure S6.** Depletion of individual APMAP interacting proteins does not affect the level of α-, β- or γ-secretases. **Figure S7.** N-linked glycosylation of APMAP1 and absence of glycosylation of APMAP2. **Figure S8.** APMAP1 predicted N-glycosylation at residue Asn160, while APMAP2 is unglycosylated. **Figure S9.** The protein levels of the APMAP interactomers clusterin, calnexin, Arg1, PTGFRN and CI-M6PR are unchanged in AD brains. (PDF 12432 kb)

Additional file 2: **Table S1.** (**a**) Summary list of 113 proteins differently expressed in APMAP ko/ko mice, compared to WT mice. (**b**) List of 2747 proteins identified by LFQ where at least 2 peptides have been detected and a minimum of 2 valid values per group (ko/ko vs wt/wt) was obtained. Displayed with a yellow background are the 105 differently expressed proteins revealed by statistical analysis (t-test: *p*-value < 0.05). (**c**) List of 8 differently expressed proteins identified in addition to (**b**) by imputing missing LFQ values with at least 2 detected peptides and a minimum of 3 valid values for at least one group (ko/ko or wt/wt; t-test: *p*-value < 0.05), in green left-censored imputated values. (XLSX 540 kb)
