# Supplementary material for: The APMAP interactome reveals new modulators of APP processing and beta-amyloid production that are altered in Alzheimer’s disease
Source: Acta Neuropathol Commun. 2019 Jan 31;7:13. doi: 10.1186/s40478-019-0660-3 (PMC6354426; doi:10.1186/s40478-019-0660-3)
Supplement: Supplementary file 1 — Figure S1. Generation of the APMAP-KO mouse line. Figure S2. Morpho-pathological characterization of APMAP-KO mice. Figure S3. Generation and selection of a CHO cell line stably overexpressing APMAP for the high-grade purification of APMAP and associated proteins. Figure S4. Mass spectrometric identification of APMAP-interacting proteins. Figure S5. Treatments of HEK-APPSwe cells with siRNAs targeting fetuin-A, serpinB3, stratifin, LAMP-1 and membralin do not affect APP-FL, APP-CTFs, or APMAP. Figure S6. Depletion of individual APMAP interacting proteins does not affect the level of α-, β- or γ-secretases. Figure S7. N-linked glycosylation of APMAP1 and absence of glycosylation of APMAP2. Figure S8. APMAP1 predicted N-glycosylation at residue Asn160, while APMAP2 is unglycosylated. Figure S9. The protein levels of the APMAP interactomers clusterin, calnexin, Arg1, PTGFRN and CI-M6PR are unchanged in AD brains. (PDF 12432 kb) [file 40478_2019_660_MOESM1_ESM.pdf]

The APMAP interactome reveals new modulators of APP processing and beta-amyloid production that are altered in Alzheimer's disease

Hermeto Gerber[1,2,3], Sebastien Mosser[1,2], Benjamin Boury-Jamot[4], Michael Stumpe[3], Alessandra Piersigilli[5,6], Christine Goepfert[5,6], Joern Dengjel[3], Urs Albrecht[3], Fulvio Magara[4] and Patrick C. Fraering[1,2*]

[1]Foundation Eclosion, CH-1228 Plan-les-Ouates, Switzerland, [2]Campus Biotech Innovation Park, CH-1202 Geneva, Switzerland, [3]Department of Biology, University of Fribourg, CH-1700 Fribourg, Switzerland, [4]Centre for Psychiatric Neuroscience, Department of Psychiatry, Lausanne University Hospital, CH-1015 Lausanne, Switzerland, [5]Institute of Animal Pathology, Vetsuisse Faculty, University of Bern, CH-3012 Bern, Switzerland, [6]School of Life Sciences, Ecole Polytechnique Fédérale de Lausanne, CH-1015 Lausanne, Switzerland.

[*]Correspondence should be addressed to Patrick C. Fraering. Phone: +41786406462; Fax: +41228801013; E-mail: fraeringpatrick@hotmail.com

SUPPLEMENTARY FIGURES

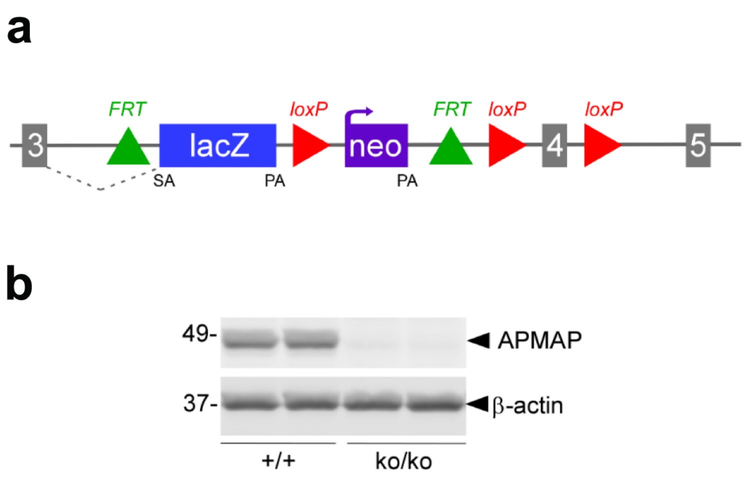

Fig.S1 Generation of the APMAP-KO mouse line. **a** Schematic representation of the knockout-first construct (KOMP Repository; www.komp.org) used for the generation of the APMAP full knockout mouse line. **b** The deleted APMAP protein expression was confirmed by Western blot analysis of whole brain extracts from 3-month-old APMAP-KO mice (ko/ko; n=2) and WT mice (+/+; n=2). β-Actin served as a protein loading control.

Gerber et. al., *Supplementary Fig.S2*

a

Skin
Mammalian gland
Heart
Tongue
Sternum / Bone marrow
Thymus
Trachea
Oesophagus
Thyroid
Salivary gland
Mandibular Lymphnodes
Lung
Stomach
Small Intestine
Large Intestine
Liver
Kidney right and left
Urinary bladder
Spleen
Pancreas
White adipose tissue
Spine (cervical, thoracal, lumbar)
Knee (joint, bones, muscles)
Head (multiple cross sections)
Brain (1 hemisphere)

**Female genital tract:**
Uterus
Ovary right and left
Vagina

**Male genital tract:**
Testis right and left
Seminal vesicle
Prostate
Preputial glands

b

	chow diet		high fat diet		significance
	n per group	grade / score	n per group	grade / score	
Lung					
leucocytic infiltrates	ko-4, wt-8	ko 1.8, wt 3.7	ko-3, wt-1	ko 3, wt 2.5	inflammatory change
Kidney					
leucocytic infiltrates, perivascular	ko-8, wt-10	ko 3.8, wt 4.1	ko-4, wt-3	ko 3.7, wt 2.8	background lesion
tubular lipidosis	ko-3, wt-3	ko 1.2, wt 1.2	ko-5, wt-2	ko-1.8, wt 1.2	incidental finding
tubular basophilia	ko-1, wt-2				background lesion
tubular proteinaceous casts	wt-2		wt-1		background lesion
basement membrane thickening	wt-1				background lesion
glomerular sklerosis	wt-1		ko-1		background lesion
mesangial cell proliferation	wt-1		wt-2		background lesion
Liver					
leucocytic infiltrates	ko-3, wt-7	ko 1.1, wt 2.5	ko-5, wt-2	ko 4.8, wt 1.2	inflammatory change
hepatic lipidosis	ko-0, wt-0		ko-5, wt-5	ko 8, wt 8	diet related
extramedullary hematopoiesis	ko-2, wt-1		wt-2		related to inflammation
Stomach					
leucocytic infiltrates	ko-4, wt-4	ko 1.3, wt 1	ko-1, wt-0	ko 1.2	inflammatory change
Oesophagus					
bacteria intracorneal			ko-1, wt-1		incidental finding
Spleen					
activated lymph follicles	ko-0, wt-1		ko-2, wt-2		background lesion
hemosiderosis	ko-6, wt-3	ko 2.7, wt 1.7	ko-1, wt-0	ko 1.2	background lesion
extramedullary hematopoiesis	ko-1, wt-1		ko-1, wt-0		related to inflammation
Salivary gland					
leucocytic infiltrates	ko-7, wt-8	ko 4, wt 4.2	ko-3, wt-1	ko 3.6, wt 1	background lesion
Lymph nodes (mandibular, mediastinal, mesenteric)					
activated lymph follicles			ko-2, wt-3		background lesion
sinus histiocytosis	ko-4, wt-7	ko 2.5, wt 3.7	ko-5, wt-12	ko 5.3, wt 1.9	background lesion
Pancreas					
leucocytic infiltrates	ko-3, wt-6	ko 1.7, wt 3.3	ko-0, wt-0		inflammatory change
Testis					
leucocytic infiltrates	ko-1, wt-2	ko-1, wt-1.17	ko-0, wt-1	wt 1	background lesion
Seminal Vesicles					
histiocytes intraluminal			ko-1		incidental finding
Preputial glands					
leucocytic infiltrates	ko-2, wt-2	ko 2.3, wt 1.5	ko-3, wt-2	ko 2.8, wt 1.6	background lesion
fibrosis	ko-0, wt-1				background lesion
cystic dilation	ko-1, wt-3		wt-3		background lesion
Skin					
leucocytic infiltrates w/wo bacteria			ko-4, wt-3	ko-3.6, wt 2.6	inflammatory change
Gingiva					
leucocytic infiltrates			ko-1	ko 0.8	incidental finding
Muscle					
leucocytic infiltrates			ko-1		incidental finding
muscle fiber regeneration			ko-1		incidental finding
Urinary bladder					
leucocytic infiltrates	wt-1				background lesion

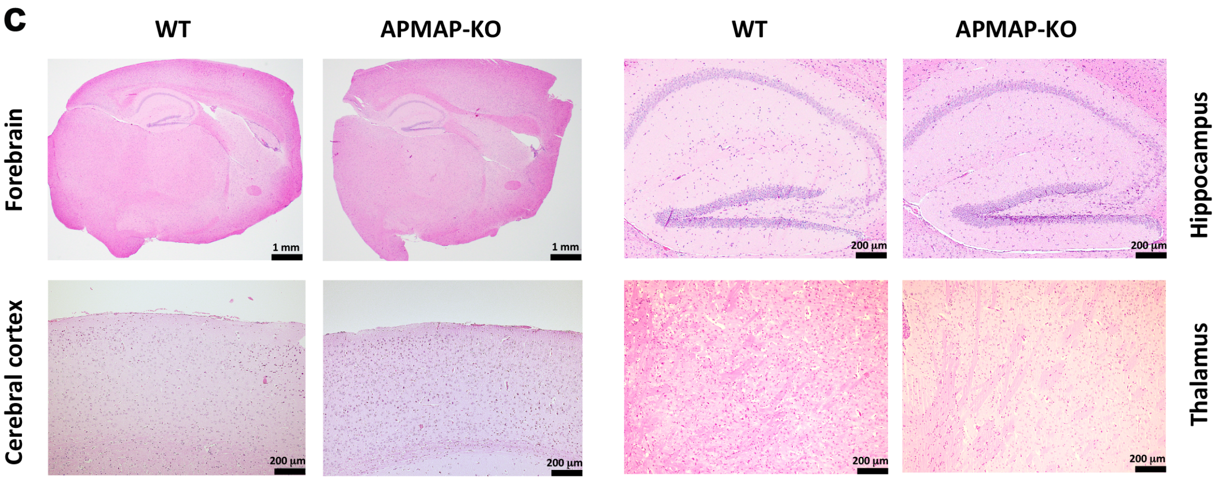

Fig.S2 Morpho-pathological characterization of APMAP-KO mice. **a** Organs and tissues analyzed to assess whether the biallelic deletion of the gene coding for APMAP induces macroscopic and/or microscopic morphological changes. **b** No significant morphological changes (graded from 0 to 6 according to their degree of severity) were recorded in the APMAP-KO groups compared to the age and sex-matched WT control animals, fed with chow or high fat diets. The phenotypic observations are not related to the specific genotype, as confirmed by the non-significant semi-quantitative histopathologic scores among the different groups, and by the intra- and intergroup low incidence. Mice on a chow diet were aged 7 to 9 months, with n=10 mice/group (equally distributed between males and females). Mice on a high fat diet were aged 4 months, with n=5 males/group. **c** APMAP deficiency does not affect morphology of neural tissues. Sagittal brain sections from 9 month-old WT and APMAP-KO mice stained with hematoxylin and eosin. Specific tissue is indicated at left of each pair of panels. Scale bars, 1 mm for brain and 200 µm for hippocampus, cerebral cortex and thalamus.

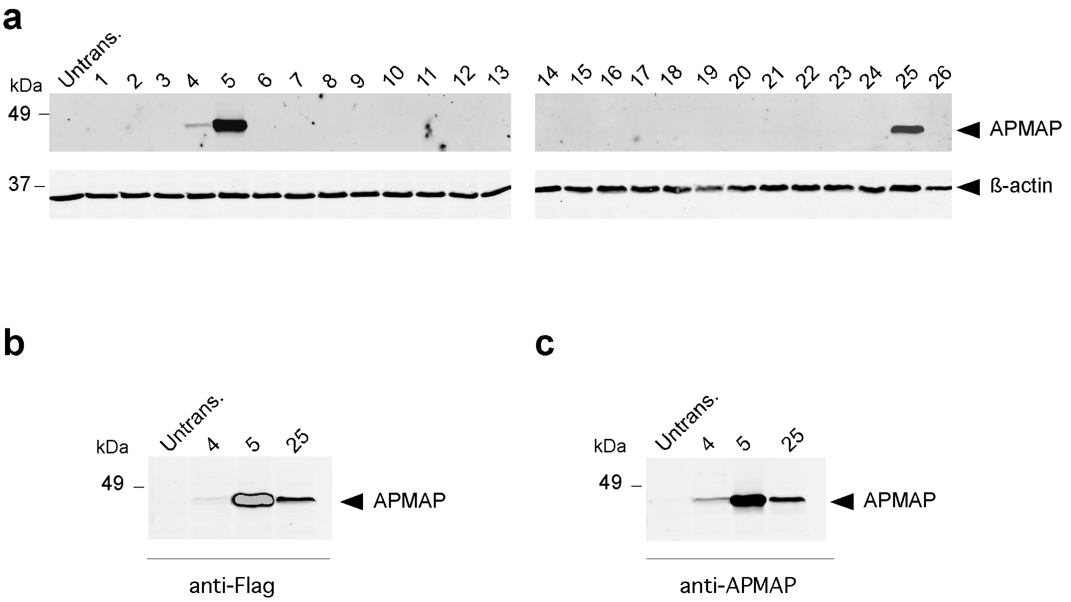

Fig.S3 Generation and selection of a CHO cell line stably overexpressing APMAP for the high-grade purification of APMAP and associated proteins. **a** Clonal selection of a CHO cell line stably overexpressing APMAP. Chinese hamster ovary adherent cells were transfected with a plasmid harboring APMAP1-Flag and selection was performed in DMEM containing 300 µg/ml Geneticin G418. After 21 days of selection, single cell cultures were inoculated into 96-well plates by limiting dilution. Of all recovered clonal cell lines, 26 were screened for APMAP expression and the highest producers were maintained in culture for further analysis. **b, c** Whole cell extracts were prepared and analyzed by Western blot with anti-Flag (**b**) or anti-APMAP (**c**) antibodies. The highest producer (clone 5) was further adapted for cultures in suspension and used for the purification of APMAP and associated proteins.

Gerber et. al., *Supplementary Fig.S4*

CP1

Q9HDC9 (100%), 46,481.5 Da
Adipocyte plasma membrane-associated protein OS=Homo sapiens GN=APMAP PE=1 SV=2
36 exclusive unique peptides, 50 exclusive unique spectra, 173 total spectra, 212/416 amino acids (51% coverage)

```
MSEADGLRQR RPLRPQVVTD DDGQAPEAKD GSSFSGRVFR VTFLMLAVSL TVPLLGAMML
LESPIDPQPL SFKEPPLLLG VLHPNTKLRQ AERLFENQLV GPESIAHIGD VMFTGTADGR
VVKLENGEIE TIARFGSGPC KTRDDEPVCG RPLGIRAGPN GTLFVADAYK GLFEVNPWKR
EVKLLLSSET PIEGKNMSFV NDLTVTQDGR KIYFTDSSSK WQRRDYLLLV MEGTDDGRLL
EYDTVTREVK VLLDQLRFPN GVQLSPAEDF VLVAETTMAR IRRVYVSGLM KGGADLFVEN
MPGFPDNIRP SSSGGYWVGM STIRPNPGFS MLDFLSERPW IKRMIFKLFS QETVMKFVPR
YSLVLELSDS GAFRRSLHDP DGLVATYISE VHEHDGHLYL GSFRSPFLCR LSLQAV
```

CP2

Q9HDC9 (100%), 46,481.5 Da
Adipocyte plasma membrane-associated protein OS=Homo sapiens GN=APMAP PE=1 SV=2
32 exclusive unique peptides, 40 exclusive unique spectra, 126 total spectra, 196/416 amino acids (47% coverage)

```
MSEADGLRQR RPLRPQVVTD DDGQAPEAKD GSSFSGRVFR VTFLMLAVSL TVPLLGAMML
LESPIDPQPL SFKEPPLLLG VLHPNTKLRQ AERLFENQLV GPESIAHIGD VMFTGTADGR
VVKLENGEIE TIARFGSGPC KTRDDEPVCG RPLGIRAGPN GTLFVADAYK GLFEVNPWKR
EVKLLLSSET PIEGKNMSFV NDLTVTQDGR KIYFTDSSSK WQRRDYLLLV MEGTDDGRLL
EYDTVTREVK VLLDQLRFPN GVQLSPAEDF VLVAETTMAR IRRVYVSGLM KGGADLFVEN
MPGFPDNIRP SSSGGYWVGM STIRPNPGFS MLDFLSERPW IKRMIFKLFS QETVMKFVPR
YSLVLELSDS GAFRRSLHDP DGLVATYISE VHEHDGHLYL GSFRSPFLCR LSLQAV
```

P12763 (100%), 38,417.4 Da
Alpha-2-HS-glycoprotein OS=Bos taurus GN=AHSG PE=1 SV=2
3 exclusive unique peptides, 3 exclusive unique spectra, 5 total spectra, 38/359 amino acids (11% coverage)

```
MKSFVLLFCL AQLWGCHSIP LDPVAGYKEP ACDDPDTEQA ALAAVDYINK HLPRGYKHTL
NQIDSVKVWP RRPTGEVYDI EIDTLETTCH VLDPTPLANC SVRQQTQHAV EGDCDIHVLK
QDGQFSVLFT KCDSSPDSAE DVRKLCPDCP LLAPLNDSRV VHAVEVALAT FNAESNGSYL
QLVEISRAQF VPLPVSVSVE FAVAATDCIA KEVVDPTKCN LLAEKQYGFC KGSVIQKALG
GEDVRVTCTL FQTQPVIPQP QPDGAEAEAP SAVPDAAGPT PSAAGPPVAS VVVGPSVVAV
PLPLHRAHYD LRHTFSGVAS VESSSGEAFH VGKTPIVGQP SIPGGPVRLC PGRIRYFKI
```

CP3

Q9HDC9 (100%), 46,481.5 Da
Adipocyte plasma membrane-associated protein OS=Homo sapiens GN=APMAP PE=1 SV=2
22 exclusive unique peptides, 25 exclusive unique spectra, 73 total spectra, 178/416 amino acids (43% coverage)

```
MSEADGLRQR RPLRPQVVTD DDGQAPEAKD GSSFSGRVFR VTFLMLAVSL TVPLLGAMML
LESPIDPQPL SFKEPPLLLG VLHPNTKLRQ AERLFENQLV GPESIAHIGD VMFTGTADGR
VVKLENGEIE TIARFGSGPC KTRDDEPVCG RPLGIRAGPN GTLFVADAYK GLFEVNPWKR
EVKLLLSSET PIEGKNMSFV NDLTVTQDGR KIYFTDSSSK WQRRDYLLLV MEGTDDGRLL
EYDTVTREVK VLLDQLRFPN GVQLSPAEDF VLVAETTMAR IRRVYVSGLM KGGADLFVEN
MPGFPDNIRP SSSGGYWVGM STIRPNPGFS MLDFLSERPW IKRMIFKLFS QETVMKFVPR
YSLVLELSDS GAFRRSLHDP DGLVATYISE VHEHDGHLYL GSFRSPFLCR LSLQAV
```

F1LQN3 (100%), 126,405.1 Da
Reticulon OS=Rattus norvegicus GN=Rtn4 PE=1 SV=1
3 exclusive unique peptides, 3 exclusive unique spectra, 3 total spectra, 29/1163 amino acids (2% coverage)

```
MEDIDQSSLV SSSTDSPPRP PPAFKYQFVT EPEDEEDEEE EEDEEEDDED LEELEVLERK
PAAGLSAAAV PPAAAAPLLD FSSDSVPPAP RGPLPAAPPA APERQPSWER SPAAPAPSLP
PAAAVLPSKL PEDDEPPARP PPPPPAGASP LAEPAAPPST PAAPKRRGSG SVDETLFALP
AASEPVIPSS AEKIMDLMEQ PGNTVSSGQE DFPSVLLETA ASLPSLSPLS TVSFKEHGYL
GNLSAVSSSE GTIEETLNEA SKELPERATN PFVNRDLAEF SELEYSEMGS SFKGSPKGES
AILVENTKEE VIVRSKDKED LVCSAALHSP QESPVGKEDR VVSPEKTMDI FNEMQMSVVA
PVREEYADFK PFEQAWEVKD TYEGSRDVLA ARANVESKVD RKCLEDSLEQ KSLGKDSEGR
NEDASFPSTP EPVKDSSRAY ITCASFTSAT ESTTANTFPL LEDHTSENKT DEKKIEERKA
QIITEKTSPK TSNPFLVAVQ DSEADYVTTD TLSKVTEAAV SNMPEGLTPD LVQEACESEL
NEATGTKIAY ETKVDLVQTS EAIQESLYPT AQLCPSFEEA EATPSPVLPD IVMEAPLNSL
LPSAGASVVQ PSVSPLEAPP PLSYDSIKLE PENPPPYEEA MNVALKALGT KEGIKEPESF
NAAVQETEAP YISIACDLIK ETKLSTEPSP DFSNYSEIAK FEKSVPEHAE LVEDSSPESE
PVDLFSDDSI PEVPQTQEEA VMLMKESLTE VSETVAQHKE ERLSASPQEL GKPYLESFQP
NLHSTKDAAS NDIPTLTKKE KISLQMEEFN TAIYSNDDLL SSKEDKIKES ETFSDSSPIE
IIDEFPTFVS AKDDSPKLAK EYTDLEVSDK SEIANIQSGA DSLPCLELPC DLSFKNIYPK
DEVHVSDEFS ENRSSVSKAS ISPSNVSALE PQTEMGSIVK SKSLTKEAEK KLPSDTEKED
RSLSAVLSAE LSKTSVVDLL YWRDIKKTGV VFGASLFLLL SLTVFSIVSV TAYIALALLS
VTISFRIYKG VIQAIQKSDE GHPFRAYLES EVAISEELVQ KYSNSALGHV NSTIKELRRL
FLVDDLVDSL KFAVLMWVFT YVGALFNGLT LLILALISLF SIPVIYERHQ VQIDHYLGLA
NKSVKDAMAK IQAKIPGLKR KAD
```

CP4

Q9HDC9 (100%), 46,481.5 Da
Adipocyte plasma membrane-associated protein OS=Homo sapiens GN=APMAP PE=1 SV=2
22 exclusive unique peptides, 27 exclusive unique spectra, 54 total spectra, 173/416 amino acids (42% coverage)

```
MSEADGLRQR RPLRPQVVTD DDGQAPEAKD GSSFSGRVFR VTFLMLAVSL TVPLLGAMML
LESPIDPQPL SFKEPPLLLG VLHPNTKLRQ AERLFENQLV GPESIAHIGD VMFTGTADGR
VVKLENGEIE TIARFGSGPC KTRDDEPVCG RPLGIRAGPN GTLFVADAYK GLFEVNPWKR
EVKLLLSSET PIEGKNMSFV NDLTVTQDGR KIYFTDSSSK WQRRDYLLLV MEGTDDGRLL
EYDTVTREVK VLLDQLRFPN GVQLSPAEDF VLVAETTMAR IRRVYVSGLM KGGADLFVEN
MPGFPDNIRP SSSGGYWVGM STIRPNPGFS MLDFLSERPW IKRMIFKLFS QETVMKFVPR
YSLVLELSDS GAFRRSLHDP DGLVATYISE VHEHDGHLYL GSFRSPFLCR LSLQAV
```

F1LQN3 (100%), 126,405.1 Da
Reticulon OS=Rattus norvegicus GN=Rtn4 PE=1 SV=1
3 exclusive unique peptides, 3 exclusive unique spectra, 5 total spectra, 41/1163 amino acids (4% coverage)

```
MEDIDQSSLV SSSTDSPPRP PPAFKYQFVT EPEDEEDEEE EEDEEEDDED LEELEVLERK
PAAGLSAAAV PPAAAAPLLD FSSDSVPPAP RGPLPAAPPA APERQPSWER SPAAPAPSLP
PAAAVLPSKL PEDDEPPARP PPPPPAGASP LAEPAAPPST PAAPKRRGSG SVDETLFALP
AASEPVIPSS AEKIMDLMEQ PGNTVSSGQE DFPSVLLETA ASLPSLSPLS TVSFKEHGYL
GNLSAVSSSE GTIEETLNEA SKELPERATN PFVNRDLAEF SELEYSEMGS SFKGSPKGES
AILVENTKEE VIVRSKDKED LVCSAALHSP QESPVGKEDR VVSPEKTMDI FNEMQMSVVA
PVREEYADFK PFEQAWEVKD TYEGSRDVLA ARANVESKVD RKCLEDSLEQ KSLGKDSEGR
NEDASFPSTP EPVKDSSRAY ITCASFTSAT ESTTANTFPL LEDHTSENKT DEKKIEERKA
QIITEKTSPK TSNPFLVAVQ DSEADYVTTD TLSKVTEAAV SNMPEGLTPD LVQEACESEL
NEATGTKIAY ETKVDLVQTS EAIQESLYPT AQLCPSFEEA EATPSPVLPD IVMEAPLNSL
LPSAGASVVQ PSVSPLEAPP PLSYDSIKLE PENPPPYEEA MNVALKALGT KEGIKEPESF
NAAVQETEAP YISIACDLIK ETKLSTEPSP DFSNYSEIAK FEKSVPEHAE LVEDSSPESE
PVDLFSDDSI PEVPQTQEEA VMLMKESLTE VSETVAQHKE ERLSASPQEL GKPYLESFQP
NLHSTKDAAS NDIPTLTKKE KISLQMEEFN TAIYSNDDLL SSKEDKIKES ETFSDSSPIE
IIDEFPTFVS AKDDSPKLAK EYTDLEVSDK SEIANIQSGA DSLPCLELPC DLSFKNIYPK
DEVHVSDEFS ENRSSVSKAS ISPSNVSALE PQTEMGSIVK SKSLTKEAEK KLPSDTEKED
RSLSAVLSAE LSKTSVVDLL YWRDIKKTGV VFGASLFLLL SLTVFSIVSV TAYIALALLS
VTISFRIYKG VIQAIQKSDE GHPFRAYLES EVAISEELVQ KYSNSALGHV NSTIKELRRL
FLVDDLVDSL KFAVLMWVFT YVGALFNGLT LLILALISLF SIPVIYERHQ VQIDHYLGLA
NKSVKDAMAK IQAKIPGLKR KAD
```

CP5

Q9HDC9 (100%), 46,481.5 Da
Adipocyte plasma membrane-associated protein OS=Homo sapiens GN=APMAP PE=1 SV=2
24 exclusive unique peptides, 30 exclusive unique spectra, 64 total spectra, 184/416 amino acids (44% coverage)

```
MSEADGLRQR RPLRPQVVTD DDGQAPEAKD GSSFSGRVFR VTFLMLAVSL TVPLLGAMML
LESPIDPQPL SFKEPPLLLG VLHPNTKLRQ AERLFENQLV GPESIAHIGD VMFTGTADGR
VVKLENGEIE TIARFGSGPC KTRDDEPVCG RPLGIRAGPN GTLFVADAYK GLFEVNPWKR
EVKLLLSSET PIEGKNMSFV NDLTVTQDGR KIYFTDSSSK WQRRDYLLLV MEGTDDGRLL
EYDTVTREVK VLLDQLRFPN GVQLSPAEDF VLVAETTMAR IRRVYVSGLM KGGADLFVEN
MPGFPDNIRP SSSGGYWVGM STIRPNPGFS MLDFLSERPW IKRMIFKLFS QETVMKFVPR
YSLVLELSDS GAFRRSLHDP DGLVATYISE VHEHDGHLYL GSFRSPFLCR LSLQAV
```

A0A061I3K8 (100%), 160,726.0 Da
Calnexin OS=Cricetulus griseus GN=H671_7g18220 PE=3 SV=1
15 exclusive unique peptides, 18 exclusive unique spectra, 22 total spectra, 109/1479 amino acids (7% coverage)

MEGKWLLCLL LVLGTVAVQA HDGHDDDMID IEDDLDDVIE EVEDSK**SKSD** **SSTPPSPK**VT
YK**APVPTGEV** **YFADSFDR**GS LSGWILSK**AK** **KDDTDDEIAK** YDGKWEVDEM KDTKLPGDKG
LVLMSRAKHH AISAKLNKPF LFDTKPLIVQ YEVNFQNGIE CGGAYVKLLS K**TSELNLDQF**
HDKTPYTIMF GPDKCGEDYK LHFIFRHKNP KTGVYEEKHA KRPDTDLKIY FTDKKTHLYT
LILNPDNSFE ILVDQYVVNS GNLLNDMTPA VNPSREIEDP EDQKPEDWDE RPKIPDPDAV
KPDDWDEDAP GKIPDEEATK PEGWLDDEPE YIPDPDADKP EDWDEDMDGE WEAPQIANPK
CESAPGCGVW QR**PLIDNPNY** **K**GKWKPPMID NPNYQGVWKP RKIPNPDFFE DLEPFKMTPF
SAIGLELWSM TSDIFFDNFI ISGDRRVVDD WANDGWGLK**K** **AADGAAEPGV** **VGH**MLEAAEE
RPWLWVVYIL TVALPVFLVI LFCCSGKKQS NAMEYK**KTDA** **AQPDVKEDEG** **KEEEKNKGDE**
EEEEEKLEEK QKSDAEEDGG TGSQDEEDRK PTAEEDEILN RSPRNRKPRR ESWDLWHLHD
TVKRNLDSAA SPQNGDQQNG YGDLFPGHKK TRREAPLGIS VSANGLPPAS PLGQPDKPPG
GDALQSGGKH SLGLDPINKK CMADSSIHLN GSSNPNEPFP LSLSKELKQE PVDDLPCMIA
GAGGSVSQSN LMPDLNLNEQ EWKELIEELN RSVPDEDMKD LFTEDFEEKK DPEPPGSATQ
TPLAQDINIK TEFSPAAFEQ EQLGSPQVRA GSAGQTFLGP SSAPVGTDSP SLGSSQTLFH
TTGQPGADNP SPNLMPASAQ AQNAQRALAS VVLPSQGPGG ASELSSAHQL QQIAAKQKRE
QMLQNPQQAA SAPAPGQLST WQQAGPSHSP LDVPYPMEKP ASPPGYKQDF TNSKLLMMPS
VNKSSPRPGG PYLQPSHSNL LSHQSPSNLN QSSVNNQGAV LDYGNTKPLS HYKADCGQGG
PGSGQSKPAL MAYLPQQLPH LSNEQNSLFL MKQKAANMPF RSLVPPGQEQ NPSNVSVPAQ
TANVGTQPPS VSVASTHNSS PYLSSQQQAA VMKQHQLLLD QQKQREQQQL QQQQQQFLQR
QHLLAEQEKQ QFQRPSAAVP GMNNLGPSNS SCPRVFPQPG TLMSMGPGHA PVSSLPSSSG
PQDRGVAQFT GSQSLPQSSL YGITSGLPQI VAQPPPQAAS AHAHIPRQTS VGQNASAPAA
YGQNSLGSAG LSQQHNKGTL PPGLTKSQVP RVSAAMGGQN ASWQHQGMPN LSSQTPGNSS
VSPFAAPSSF HMQQAHLKIP GQQFSQAMPS RPMAPMSSAG AAGSMLPPVS TQQRNSAPAP
APPQAAPQQG LPGLSPAGPE LGAFSQSSTS QMNSRAGLHC TQAYPVRTMG QELPFAFSGQ
PGSSGLSSVA GHTDLIDSLL KNRTSEEWIN ELDDLLGSQ

A0A061IM78 (100%), 43,787.5 Da
Lysosome-associated membrane glycoprotein 1 OS=Cricetulus griseus GN=H671_1g1762 PE=4 SV=1
2 exclusive unique peptides, 2 exclusive unique spectra, 2 total spectra, 23/407 amino acids (6% coverage)

MAAPGAPRSL LLLLLAGLAH GASALFVVKD SNGTACIMAN FSASFFTIYE TGHGSKNSTF
ELPSSAEVLN SNSSCGRENV SEPILTIAFG SGYLLTLNFT RNATRYSVQD MYFAYNLSDT
QHFLNASNK**G** **IHSVDSSTDI** **K**ADINKTYRC LSAIQVHMGN VTVTLSDATI QAYLLNSNFS
KEETRCTQDG PSPTTVPPSP SPPLVPTNPT VIKYNVTGEN GTCLLASMAL QMNITYMKKD
NMTVTRALNI SPNDTASGSC SPHVVTLTVE SKNSILDLKF GMNGSSSLFF LQEVRLNMTL
PDANVSSLMA SNQSLR**ALQA** **TVGNSYK**CNT EEHIFVTKEF SLNVFSVQVQ AFKVESDRFG
SVEECMQDGN NMLIPIAVGG ALAGLVLIVL IAYLIGRKRS HAGYQTI

G3H7X9 (100%), 68,661.3 Da
Nicastrin OS=Cricetulus griseus GN=I79_006475 PE=4 SV=1
3 exclusive unique peptides, 3 exclusive unique spectra, 3 total spectra, 35/615 amino acids (6% coverage)

MVLLEGKLFN RAVMEKLKGR TSRIAGLAVT LAKPNSTSSF SPSVQCPNDG FGIYSNSYGS
EFAHCKQTLW NELGNGLAYE DFSFPIFLLE DENETKVIKQ CYQDHNLAQN GSAPRFPLCA
MQLFSHMHAV ISTATCMRRS FIQSTFSINP EIVCDPLSDY NVWSMLKPIN TSGALEPDDR
VVVAATRLDS RSFFWNVAPG AESAVASYVT QLAAAEALHK **ASDVATLPR**N VMFVFFQGET
FDYIGSSRMV YDMENGKFPV RLENIDSFVE LGQVALRTSL DLWMHTDPMS QKNESVKNQV
EDLLVTLEKS GAGVPEVVLR RQNQSQALPP SSLQRFLRAR NISGVVLADH SGSFHNRYYQ
SIYDTAENIN VTYPER**QSPE** **EDLNFVTDTA** **KALANVATVL** **AR**ALYELAGG TNFSDSIQAD
PQTVTRLLYG FLVRANNSWF QSILRQDLRS YLDDGPLQHY IAVTSPTNTT YVVQYALANL
TGKVTNLTRE QCQDPSKVPN ESKALYDYSW VQGPLNSNKT ERLPRCVRST VRLARALSPA
FELSQWGSTE YSTWAESRWK DIQARIFLVA SKELEFITLT VGFGILIFSL IVTYCINAKA
DVLFVAPREP GAVSY

F1LQN3 (100%), 126,405.1 Da
Reticulon OS=Rattus norvegicus GN=Rtn4 PE=1 SV=1
2 exclusive unique peptides, 2 exclusive unique spectra, 2 total spectra, 22/1163 amino acids (2% coverage)

MEDIDQSSLV SSSTDSPPRP PPAFKYQFVT EPEDEEDEEE EEDEEEDDED LEELEVLERK
PAAGLSAAAV PPAAAAPLLD FSSDSVPPAP RGPLPAAPPA APERQPSWER SPAAPAPSLP
PAAAVLPSKL PEDDEPPARP PPPPPAGASP LAEPAAPPST PAAPKRRGSG SVDETLFALP
AASEPVIPSS AEKIMDLMEQ PGNTVSSGQE DFPSVLLETA ASLPSLSPLS TVSFKEHGYL
GNLSAVSSSE GTIEETLNEA SKELPERATN PFVNRDLAEF SELEYSEMGS SFKGSPKGES
AILVENTKEE VIVRSKDKED LVCSAALHSP QESPVGKEDR VVSPEKTMDI FNEMQMSVVA
PVREEYADFK PFEQAWEVKD TYEGSRDVLA ARANVESKVD RKCLEDSLEQ KSLGKDSEGR
NEDASFPSTP EPVKDSSRAY ITCASFTSAT ESTTANTFPL LEDHTSENKT DEKKIEERKA
QIITEKTSPK TSNPFLVAVQ DSEADYVTTD TLSKVTEAAV SNMPEGLTPD LVQEACESEL
NEATGTKIAY ETKVDLVQTS EAIQESLYPT AQLCPSFEEA EATPSPVLPD IVMEAPLNSL
LPSAGASVVQ PSVSPLEAPP PLSYDSIKLE PENPPPYEEA MNVALKALGT KEGIKEPESF
NAAVQETEAP YISIACDLIK ETKLSTEPSP DFSNYSEIAK FEKSVPEHAE LVEDSSPESE
PVDLFSDDSI PEVPQTQEEA VMLMKESLTE VSETVAQHKE ERLSASPQEL GKPYLESFQP
NLHSTKDAAS NDIPTLTKKE KISLQMEEFN TAIYSNDDLL SSKEDKIKES ETFSDSSPIE
IIDEFPTFVS AKDDSPKLAK EYTDLEVSDK SEIANIQSGA DSLPCLELPC DLSFKNIYPK
DEVHVSDEFS ENRSSVSKAS ISPSNVSALE PQTEMGSIVK SKSLTKEAEK KLPSDTEKED
RSLSAVLSAE LSKTSVVDLL YWRDIKKTGV VFGASLFLLL SLTVFSIVSV TAYIALALLS
VTISFRIYKG VIQAIQK**SDE** **GHPFR**AYLES EVAISEELVQ K**YSNSALGHV** **NSTIK**ELRRL
FLVDDLVDSL KFAVLMWVFT YVGALFNGLT LLILALISLF SIPVIYERHQ VQIDHYLGLA
NKSVKDAMAK IQAKIPGLKR KAD

CP6

Q9HDC9 (100%), 46,481.5 Da
Adipocyte plasma membrane-associated protein OS=Homo sapiens GN=APMAP PE=1 SV=2
31 exclusive unique peptides, 37 exclusive unique spectra, 59 total spectra, 204/416 amino acids (49% coverage)

```
MSEADGLRQR RPLRPQVVTD DDGQAPEAKD GSSFSGRVFR VTFLMLAVSL TVPLLGAMML
LESPIDPQPL SFKEPPLLLG VLHPNTKLRQ AERLFENQLV GPESIAHIGD VMFTGTADGR
VVKLENGEIE TIARFGSGPC KTRDDEPVCG RPLGIRAGPN GTLFVADAYK GLFEVNPWKR
EVKLLLSSET PIEGKNMSFV NDLTVTQDGR KIYFTDSSSK WQRRDYLLLV MEGTDDGRLL
EYDTVTREVK VLLDQLRFPN GVQLSPAEDF VLVAETTMAR IRRVYVSGLM KGGADLFVEN
MPGFPDNIRP SSSGGYWVGM STIRPNPGFS MLDFLSERPW IKRMIFKLFS QETVMKFVPR
YSLVLELSDS GAFRRSLHDP DGLVATYISE VHEHDGHLYL GSFRSPFLCR LSLQAV
```

A0A061IM78 (100%), 43,787.5 Da
Lysosome-associated membrane glycoprotein 1 OS=Cricetulus griseus GN=H671_1g1762 PE=4 SV=1
2 exclusive unique peptides, 2 exclusive unique spectra, 2 total spectra, 23/407 amino acids (6% coverage)

```
MAAPGAPRSL LLLLLAGLAH GASALFVVKD SNGTACIMAN FSASFFTIYE TGHGSKNSTF
ELPSSAEVLN SNSSCGRENV SEPILTIAFG SGYLLTLNFT RNATRYSVQD MYFAYNLSDT
QHFLNASNKG IHSVDSSTDI KADINKTYRC LSAIQVHMGN VTVTLSDATI QAYLLNSNFS
KEETRCTQDG PSPTTVPPSP SPPLVPTNPT VIKYNVTGEN GTCLLASMAL QMNITYMKKD
NMTVTRALNI SPNDTASGSC SPHVVTLTVE SKNSILDLKF GMNGSSSLFF LQEVRLNMTL
PDANVSSLMA SNQSLRALQA TVGNSYKCNT EEHIFVTKEF SLNVFSVQVQ AFKVESDRFG
SVEECMQDGN NMLIPIAVGG ALAGLVLIVL IAYLIGRKRS HAGYQTI
```

A0A061I3K8 (100%), 160,726.0 Da
Calnexin OS=Cricetulus griseus GN=H671_7g18220 PE=3 SV=1
21 exclusive unique peptides, 24 exclusive unique spectra, 30 total spectra, 141/1479 amino acids (10% coverage)

```
MEGKWLLCLL LVLGTVAVQA HDGHDDDMID IEDDLDDVIE EVEDSKSKSD SSTPPSPKVT
YKAPVPTGEV YFADSFDRGS LSGWILSKAK KDDTDDEIAK YDGKWEVDEM KDTKLPGDKG
LVLMSRAKHH AISAKLNKPF LFDTKPLIVQ YEVNFQNGIE CGGAYVKLLS KTSELNLDQF
HDKTPYTIMF GPDKCGEDYK LHFIFRHKNP KTGVYEEKHA KRPDTDLKIY FTDKKTHLYT
LILNPDNSFE ILVDQYVVNS GNLLNDMTPA VNPSREIEDP EDQKPEDWDE RPKIPDPDAV
KPDDWDEDAP GKIPDEEATK PEGWLDDEPE YIPDPDADKP EDWDEDMDGE WEAPQIANPK
CESAPGCGVW QRPLIDNPNY KGKWKPPMID NPNYQGVWKP RKIPNPDFFE DLEPFKMTPF
SAIGLELWSM TSDIFFDNFI ISGDRRVVDD WANDGWGLKK AADGAAEPGV VGHMLEAAEE
RPWLWVVYIL TVALPVFLVI LFCCSGKKQS NAMEYKKTDA AQPDVKEDEG KEEEKNKGDE
EEEEEKLEEK QKSDAEEDGG TGSQDEEDRK PTAEEDEILN RSPRNRKPRR ESWDLWHLHD
TVKRNLDSAA SPQNGDQQNG YGDLFPGHKK TRREAPLGIS VSANGLPPAS PLGQPDKPPG
GDALQSGGKH SLGLDPINKK CMADSSIHLN GSSNPNEPFP LSLSKELKQE PVDDLPCMIA
GAGGSVSQSN LMPDLNLNEQ EWKELIEELN RSVPDEDMKD LFTEDFEEKK DPEPPPGSATQ
TPLAQDINIK TEFSPAAFEQ EQLGSPQVRA GSAGQTFLGP SSAPVGTDSP SLGSSQTLFH
TTGQPGADNP SPNLMPASAQ AQNAQRALAS VVLPSQGPGG ASELSSAHQL QQIAAKQKRE
QMLQNPQQAA SAPAPGQLST WQQAGPSHSP LDVPYPMEKP ASPPGYKQDF TNSKLLMMPS
VNKSSPRPGG PYLQPSHSNL LSHQSPSNLN QSSVNNQGAV LDYGNTKPLS HYKADCGQGG
PGSGQSKPAL MAYLPQQLPH LSNEQNSLFL MKQKAANMPF RSLVPPGQEQ NPSNVSVPAQ
TANVGTQPPS VSVASTHNSS PYLSSQQQAA VMKQHQLLLD QQKQREQQQL QQQQQQFLQR
QHLLAEQEKQ QFQRPSAAVP GMNNLGPSNS SCPRVFPQPG TLMSMGPGHA PVSSLPSSSG
PQDRGVAQFT GSQSLPQSSL YGITSGLPQI VAQPPPQAAS AHAHIPRQTS VGQNASAPAA
YGQNSLGSAG LSQQHNKGTL PPGLTKSQVP RVSAAMGGQN ASWQHQGMPN LSSQTPGNSS
VSPFAAPSSF HMQQAHLKIP GQQFSQAMPS RPMAPMSSAG AAGSMLPPVS TQQRNSAPAP
APPQAAPQQG LPGLSPAGPE LGAFSQSSTS QMNSRAGLHC TQAYPVRTMG QELPFAFSGQ
PGSSGLSSVA GHTDLIDSLL KNRTSEEWIN ELDDLLGSQ
```

G3H7X9 (100%), 68,661.3 Da
Nicastrin OS=Cricetulus griseus GN=I79_006475 PE=4 SV=1
4 exclusive unique peptides, 4 exclusive unique spectra, 4 total spectra, 44/615 amino acids (7% coverage)

```
MVLLEGKLFN RAVMEKLKGR TSRIAGLAVT LAKPNSTSSF SPSVQCPNDG FGIYSNSYGS
EFAHCKQTLW NELGNGLAYE DFSFPIFLLE DENETKVIKQ CYQDHNLAQN GSAPRFPLCA
MQLFSHMHAV ISTATCMRRS FIQSTFSINP EIVCDPLSDY NVWSMLKPIN TSGALEPDDR
VVVAATRLDS RSFFWNVAPG AESAVASYVT QLAAAEALHK ASDVATLPRN VMFVFFQGET
FDYIGSSRMV YDMENGKFPV RLENIDSFVE LGQVALRTSL DLWMHTDPMS QKNESVKNQV
EDLLVTLEKS GAGVPEVVLR RQNQSQALPP SSLQRFLRAR NISGVVLADH SGSFHNRYYQ
SIYDTAENIN VTYPERQSPE EDLNFVTDTA KALANVATVL ARALYELAGG TNFSDSIQAD
PQTVTRLLYG FLVRANNSWF QSILRQDLRS YLDDGPLQHY IAVTSPTNTT YVVQYALANL
TGKVTNLTRE QCQDPSKVPN ESKALYDYSW VQGPLNSNKT ERLPRCVRST VRLARALSPA
FELSQWGSTE YSTWAESRWK DIQARIFLVA SKELEFITLT VGFGILIFSL IVTYCINAKA
DVLFVAPREP GAVSY
```

G3H902 (100%), 93,714.9 Da
Prostaglandin F2 receptor negative regulator OS=Cricetulus griseus GN=I79_006863 PE=4 SV=1
2 exclusive unique peptides, 2 exclusive unique spectra, 4 total spectra, 20/830 amino acids (2% coverage)

MASVCRGRVV RVPSGSLVRV VGTELVIPCN VSDYDGPIEQ NFDWSFSSSG SSFLELASTW
EPGFPAQLYR ERLQRGDILL RRTANDAVEL HIKNVQPSDQ GHYKCSTPST DATVQGNYED
TVQVKVLADA LVVGPSSRPP PGLSLREGEP FELRCITSTM SPLHTHLALR WELHRGPVHR
SILALSHEGR FHPGPGYEQR YHSGDVR**LDT** **AGSDAYR**LSV ARALSADQGS YRCVVSEWIT
EQGNWQEIQE KAVEVATVVI QPTALQLAVP RTVSVTEGKD LDLSCNITTD RVDDVRPEVT
WYFKKTPDAS LLASHMLARL DRDSLVHSSP HVALSHVDTR SYHLLVRDAS KENSGYYLCL
VALWAPGHNR SWHKVAEAMS APSSVSVTWL EPEYQVYLNA SKVPGFSDDP TELQCRVIDM
KRVEAGVRLT VSWYYRMNRR IGDEVVASEL LAVMDGDWTL RYGERSKQR**A** **QSGEFIFSK**E
HTDTFSFRIQ RTTEEDRGNY YCVVSAWTRQ RNNSWVKSKD VFSKPVNIFW ASEDSVLVVR
ARQPKPFFAA GNTFEMTCKV SSKNIKSPRY SVLITAEKPV GDLTSPNETK YIISLDQDSV
VKLENWTDAS RVDGVVLEKV QEDEFRYRMY QTQVSDAGLY RCMVTAWSPI GGSLWREAAT
SLSNPIEIDF QTSDDMAFDV SWFAVHSFGL DKDPVLLSSL DRKGVVTTGQ RDWKSTLSLE
RVSVLEFLLQ VHGSEDQDFG NYYCSVTPWV RSPTGSWQRE AEIHSRPIFI TVKMDVLNAF
KYPLLIGVGL STVIGLLSCL IGYCSSHWCC KKEVRETRRE RRRLMSMEMD

CP7

Q9HDC9 (100%), 46,481.5 Da
Adipocyte plasma membrane-associated protein OS=Homo sapiens GN=APMAP PE=1 SV=2
19 exclusive unique peptides, 22 exclusive unique spectra, 26 total spectra, 146/416 amino acids (35% coverage)

MSEADGLR**QR** **RPLRPQVVTD** **DDGQAPEAKD** **GSSFSGR**VFR VTFLMLAVSL TVPLLGAMML
LESPIDPQPL SFK**EPPLLLG** **VLHPNTK**LRQ AER**LFENQLV** **GPESIAH**IGD VMFTGTADGR
VVK**LENGEIE** **TIAR**FGSGPC K**TRDDEPVCG** **RPLGIR**AGPN GTLFVADAYK GLFEVNPWKR
EVK**LLLSSET** **PIEGK**NMSFV NDLTVTQDGR **KIYFTDSSSK** WQR**RDYLLLV** **MEGTDDGRLL**
EYDTVTREVK VLLDQLRFPN GVQLSPAEDF VLVAETTMAR IRR**VYVSGLM** **K**GGADLFVEN
MPGFPDNIRP SSSGGYWVGM STIRPNPGFS MLDFLSERPW IKRMIFK**LFS** **QETVMK**FVPR
YSLVLELSDS GAFRRSLHDP DGLVATYISE VHEHDGHLYL GSFRSPFLCR LSLQAV

A0A061I3K8 (100%), 160,726.0 Da
Calnexin OS=Cricetulus griseus GN=H671_7g18220 PE=3 SV=1
10 exclusive unique peptides, 12 exclusive unique spectra, 14 total spectra, 115/1479 amino acids (8% coverage)

MEGKWLLCLL LVLGTVAVQA HDGHDDDMID IEDDLDDVIE EVEDSKSK**SD** **SSTPPSPK**VT
YK**APVPTGEV** **YFADSFDR**GS LSGWILSKAK **KDDTDDEIAK** YDGKWEVDEM KDTKLPGDKG
LVLMSRAKHH AISAKLNKPF LFDTKPLIVQ YEVNFQNGIE CGGAYVKLLS K**TSELNLDQF**
HDKTPYTIMF **GPDK**CGEDYK LHFIFRHKNP KTGVYEEKHA KRPDTDLKIY FTDKKTHLYT
LILNPDNSFE ILVDQYVVNS GNLLNDMTPA VNPSREIEDP EDQKPEDWDE RPKIPDPDAV
KPDDWDEDAP GKIPDEEATK PEGWLDDEPE YIPDPDADKP EDWDEDMDGE WEAPQIANPK
CESAPGCGVW QR**PLIDNPNY** **K**GKWKPPMID NPNYQGVWKP RKIPNPDFFE DLEPFKMTPF
SAIGLELWSM TSDIFFDNFI ISGDRRVVDD WANDGWGLKK **AADGAAEPGV** **VGH**MLEAAEE
RPWLWVVYIL TVALPVFLVI LFCCSGKKQS NAMEYK**KTDA** **AQPDVKEDEG** **KEEEKNKGDE**
EEEEEKLEEK QKSDAEEDGG TGSQDEEDRK PTAEEDEILN RSPRNRKPRR ESWDLWHLHD
TVKRNLDSAA SPQNGDQQNG YGDLFPGHKK TRREAPLGIS VSANGLPPAS PLGQPDKPPG
GDALQSGGKH SLGLDPINKK CMADSSIHLN GSSNPNEPFP LSLSKELKQE PVDDLPCMIA
GAGGSVSQSN LMPDLNLNEQ EWKELIEELN RSVPDEDMKD LFTEDFEEKK DPEPPGSATQ
TPLAQDINIK TEFSPAAFEQ EQLGSPQVRA GSAGQTFLGP SSAPVGTDSP SLGSSQTLFH
TTGQPGADNP SPNLMPASAQ AQNAQRALAS VVLPSQGPGG ASELSSAHQL QQIAAKQKRE
QMLQNPQQAA SAPAPGQLST WQQAGPSHSP LDVPYPMEKP ASPPGYKQDF TNSKLLMMPS
VNKSSPRPGG PYLQPSHSNL LSHQSPSNLN QSSVNNQGAV LDYGNTKPLS HYKADCGQGG
PGSGQSKPAL MAYLPQQLPH LSNEQNSLFL MKQKAANMPF RSLVPPGQEQ NPSNVSVPAQ
TANVGTQPPS VSVASTHNSS PYLSSQQQAA VMKQHQLLLD QQKQREQQQL QQQQQQFLQR
QHLLAEQEKQ QFQRPSAAVP GMNNLGPSNS SCPRVFPQPG TLMSMGPGHA PVSSLPSSSG
PQDRGVAQFT GSQSLPQSSL YGITSGLPQI VAQPPPQAAS AHAHIPRQTS VGQNASAPAA
YGQNSLGSAG LSQQHNKGTL PPGLTKSQVP RVSAAMGGQN ASWQHQGMPN LSSQTPGNSS
VSPFAAPSSF HMQQAHLKIP GQQFSQAMPS RPMAPMSSAG AAGSMLPPVS TQQRNSAPAP
APPQAAPQQG LPGLSPAGPE LGAFSQSSTS QMNSRAGLHC TQAYPVRTMG QELPFAFSGQ
PGSSGLSSVA GHTDLIDSLL KNRTSEEWIN ELDDLLGSQ

G3HGQ1 (100%), 186,663.3 Da
Cation-independent mannose-6-phosphate receptor OS=Cricetulus griseus GN=I79_009791 PE=4 SV=1
14 exclusive unique peptides, 14 exclusive unique spectra, 14 total spectra, 152/1700 amino acids (9% coverage)

```
MPLVCSLYVH PLPLLIGDSL LRSSSRSLLE FNTTTDCQPS DSQHRTQTSI TFLCGKTLGT
PEFVTATDCV HYFEWRTTAA CKKDIFKADK EVPCYAFDDK LKKHDLNPLI KLSGGYLVDD
SDPETSLFIN VCRDIDSLRD PSTQLRACPA GTAACLLKGD QAFDVGRPKE GLKLVSKDRL
VLSYVKEEAE KPDFCNGHSP AVTITFVCPS ERREGTIPKL TASSNCRYEV EWITEYACHR
DYLESETCSL SSEQHDIAID LSPLAQQGGS SYVSDGREYT FYINVCGNAT VPLCNLKESA
VCQVKKADST QVKIAGRHQN QTLRYSDGDL TLIYSGGDEC SSGFQRMSVI NFECNKTAGN
DGRGEPVFTG EVDCTYFFTW DTRYACVKEK EDLLCGVSDG KKRYDLSVLA RHSESEKNWE
AVDGDQAESE KKYFFINVCH RVLQEGKARS CPEDAAVCAV DKNGSKNLGK FVSSPTSEKG
YIQLSYSDGD DCGSDKKITT NITLVCKPGD LESAPVLRTA GPDGCFYEFE WHTAAACVLS
KTEGENCTVF DAQAGFSFDL SPLTKKNGAY KVGTEKYDFY INVCGPVSVD LCQPNSGACQ
VAKRQEEDNS TYNFRWYTSY ACPEEPLECM VTDPSMMEQY DLSSLVKSEG RSGGNWYAME
NSREHVTRRK YYINVCRPLN PVPGCDRYAA ACQMRYENSE GSLAETVSIS NLGVAKTGPV
VEESGSLLLE YVNGSACTTS DGRLTTYSTR IHLVCGRGSL NTHPIFTYNW ECVVSFLWNT
EAACPIQTIT DTDQACSIRD PNSGFVFNLS PLNRSQGYVV PGIGKNFVFN ICGAMPECGT
IAGNPAFGCE AGTQTELESL KPERPVGMEK SLQLSTEGFL TLTYKGSSPS DKGTAFIIRF
ICNDDIYPGT AKFLHQDIDS TRGIRNTFFE FETALACVPS RVDCQVTDPA GNEYDLSALS
MAGKPWTAVD TSVDGKKRSF YLSVCTPLPY IPGCHGSAVG SCMVLPAEDR GLNLGVVQIS
PQADANGSLS ILYVNGDKCG NQRFSTRIIF ECAQTLGSPV FQFVKDCEYM FVWRTVEACP
VVREEGDNCQ VKDPRHGNFY DLKPLALSDT IITAGEYTYY FRVCGKLSSN VCHVRDGSKV
VSSCQEKKGS QGFQKVAGLL TQKLTYENGL LKMNYTGGDT CHQVYQRSTT IYFYCDRSTQ
KPVFLRETTD CSYLFEWRTQ YACPPFNVTE CSTQDGAGNS IDLSSLSRYS DNWEAVTRTG
ATEHYLINIC KSLSPRAGGG EPCPPEAAVC LLDGSKPLNL GKVRDGPQWT GGVTVLKYVD
GDLCPDQIRK RSTIIRFTCS ESQVSSRPLF ISAVQDCEYT FSWPTPAACP VKNSTHDDCQ
VTNPSTGHLF DLSSLSGKAG VTVSYSEKGL VYMSICGENE NCGAGVGACF GQTRISVGKA
SKRLSYVDQV LQLVYEEGSL CPSKSGLRYK SVISFVCRPE AGPTNRPMLI SLDKQTCTLF
FSWHTPLACE QVTECTVRNG SSIIDLSPLI HRTGGYEAYD ESEDGTSDTT PDFYINICQP
LNPMHGVPCP AGAAVCKVPV NGPPIDIGRV TGPPIFNPVA NEVYLNFESS TPCLADKHMN
YTSLIAFHCK RGVSMGTPKL IRTNDCDFVF EWETPIVCPD EVKTQGCSVT DEQLLYSFNL
TSLSTSTFKA RLPSIARGSD
```

P10909 (100%), 52,495.0 Da
Clusterin OS=Homo sapiens GN=CLU PE=1 SV=1
2 exclusive unique peptides, 2 exclusive unique spectra, 2 total spectra, 19/449 amino acids (4% coverage)

```
MMKTLLLFVG LLLTWESGQV LGDQTVSDNE LQEMSNQGSK YVNKEIQNAV NGVKQIKTLI
EKTNEERKTL LSNLEEAKKK KEDALNETRE SETKLKELPG VCNETMMALW EECKPCLKQT
CMKFYARVCR SGSGLVGRQL EEFLNQSSPF YFWMNGDRID SLLENDRQQT HMLDVMQDHF
SRASSIIDEL FQDRFFTREP QDTYHYLPFS LPHRRPHFFF PKSRIVRSLM PFSPYEPLNF
HAMFQPFLEM IHEAQQAMDI HFHSPAFQHP PTEFIREGDD DRTVCREIRH NSTGCLRMKD
QCDKCREILS VDCSTNNPSQ AKLRRELDES LQVAERLTRK YNELLKSYQW KMLNTSSLLE
QLNEQFNWVS RLANLTQGED QYYLRVTTVA SHTSDSDVPS GVTEVVVKLF DSDPITVTVP
VEVSRKNPKF METVAEKALQ EYRKKHREE
```

F1LPC5 (100%), 63,343.8 Da
Protein Tmem259 OS=Rattus norvegicus GN=Tmem259 PE=1 SV=2
3 exclusive unique peptides, 3 exclusive unique spectra, 3 total spectra, 30/574 amino acids (5% coverage)

```
MSEHAAAPGP GPNGGGGGGA APVRGPRGPN LNPNPLINVR DRLFHALFFK MAVTYSRLFP
PAFRRLFEFF VLLKALFVLF VLAYIHIVFS RSPINCLEHV RDRWPREGVL RVEVRHNSSR
APVILQFCDG GLGGLDLEPG GLELEEEELS VEMFTNSSIK FELDIEPKVF KPPGGADALN
DSQDFPFPET PAKAWPQDEY IVEYSLEYGF LRLSQATRQR LSIPVMVVTL DPTREQCFGD
RFSRLLLDEF LGYDDVLMSS VKGLAENEEN KGFLRNVVSG EHYRFVSMWM ARTSYLAAFV
IMVIFTLSVS MLLRYSHHQI FVFIVDLLQM LEMNMAIAFP AAPLLTVILA LVGMEAIMSE
FFNDTTTAFY IILIVWLADQ YDAICCHTNT SKRHWLRFFY LYHFAFYAYH YRFNGQYSSL
ALVTSWLFIQ HSMIYFFHHY ELPAILQQIR IQEMLLQTPP LGPGTPTALP DDLNNNSGSP
ATPDPSPPLA LGPSSSPAPT GGASGPGSLG AGASVSGSDL GWVAETAAII SDASFLSGLS
ASLLERRPAA PSAPDSARPD PGASLEDAPA PAGS
```

CP8

Q9HDC9 (100%), 46,481.5 Da
Adipocyte plasma membrane-associated protein OS=Homo sapiens GN=APMAP PE=1 SV=2
8 exclusive unique peptides, 10 exclusive unique spectra, 10 total spectra, 100/416 amino acids (24% coverage)

```
MSEADGLRQR RPLRPQVVTD DDGQAPEAKD GSSFSGRVFR VTFLMLAVSL TVPLLGAMML
LESPIDPQPL SFKEPPLLLG VLHPNTKLRQ AERLFENQLV GPESIAHIGD VMFTGTADGR
VVKLENGEIE TIARFGSGPC KTRDDEPVCG RPLGIRAGPN GTLFVADAYK GLFEVNPWKR
EVKLLLSSET PIEGKNMSFV NDLTVTQDGR KIYFTDSSSK WQRRDYLLLV MEGTDDGRLL
EYDTVTREVK VLLDQLRFPN GVQLSPAEDF VLVAETTMAR IRRVYVSGLM KGGADLFVEN
MPGFPDNIRP SSSGGYWVGM STIRPNPGFS MLDFLSERPW IKRMIFKLFS QETVMKFVPR
YSLVLELSDS GAFRRSLHDP DGLVATYISE VHEHDGHLYL GSFRSPFLCR LSLQAV
```

A0A096P063 (100%), 34,826.0 Da
Arginase OS=Papio anubis GN=ARG1 PE=3 SV=1
2 exclusive unique peptides, 2 exclusive unique spectra, 2 total spectra, 22/323 amino acids (7% coverage)

```
MSAKSRTIGI IGAPFSKGQP RGGVEEGPTV LRKAGLLEKL EEQECDVKDY GDLSFADIPN
DSPFQIVKNP RSVGKATEQL AGKVAEVKKN GRISLVLGGD HRSLAIGSIS GHASVHPDLG
VIWVDAHTDI NTPLTTTTGN LHGQPVSFLL KELKGKIPDV PGFSWVTPCI SAKDIVYIGL
RDVDPGEHYI LKTLGIKYFS MTEVDRLGIG KVMEETLSYL LGRKKRPIHL SFDVDGLDPS
FTPATGTPVV GGLSYREGLY ITEEIYKTGL LSGLDIMEVN PSLGKTPEEV TRTVNTAVAI
ILACFGVARE GNHKPIDYLN PPK
```

G3HZE5 (100%), 69,919.3 Da
Heat shock 70 kDa protein 1A OS=Cricetulus griseus GN=I79_016446 PE=3 SV=1
6 exclusive unique peptides, 6 exclusive unique spectra, 14 total spectra, 81/641 amino acids (13% coverage)

```
MAKSTAIGID LGTTYSCVGV FQHGKVEIIA NDQGNRTTPS YVAFTDTERL IGDAAKNQVA
LNPQNTVFDA KRLIGRKFGD AVVQADMKHW PFQVVNDGDK PKVQVSYKGE TRAFYPEEIS
SMVLTKMKEV AEAYLGHPVT NAVITVPAYF NDSQRQATKD AGVIAGLNVL RIINEPTAAA
IAYGLDRSGK GERNVLIFDL GGGTFDVSIL TIDDGIFEVK ATAGDTHLGG EDFDNRLVSH
FVEEFKRKHK KDISQNKRAV RRLRTACERA KRTLSSSTQA NLEIDSLFEG IDFYTSITRA
RFEELCADLF RGTLEPVEKS LRDAKMDKAK IHDIVLVGGS TRIPKVQKLL QDFFNGRDLN
KSINPDEAVA YGAAVQAAIL MGDKSENVQD LLLLDVAPLS LGLETAGGVM TALIKRNSTI
PTKQTQTFTT YSDNQPGVLI QVYEGERAMT RDNNLLGRFE LSGIPPAPRG VPQIEVTFDI
DANGILNVTA TDKSTGKANK ITITNDKGRL SKEEIERMVQ EAERYKAEDE VQRERVAAKN
ALESYAFNMK SAVEDEGLKG KISEADRKKV LDGCQEVISW LDANTLADKE EFVHKRQELE
RVCGPIVSGL YQGAGAPGAG GFGAQAPKGG SGSGPTIEEV D
```

P29508 (100%), 44,565.9 Da
Serpin B3 OS=Homo sapiens GN=SERPINB3 PE=1 SV=2
22 exclusive unique peptides, 27 exclusive unique spectra, 28 total spectra, 194/390 amino acids (50% coverage)

```
MNSLSEANTK FMFDLFQQFR KSKENNIFYS PISITSALGM VLLGAKDNTA QQIKKVLHFD
QVTENTTGKA ATYHVDRSGN VHHQFQKLLT EFNKSTDAYE LKIANKLFGE KTYLFLQEYL
DAIKKFYQTS VESVDFANAP EESRKKINSW VESQTNEKIK NLIPEGNIGS NTTLVLVNAI
YFKGQWEKKF NKEDTKEEKF WPNKNTYKSI QMMRQYTSFH FASLEDVQAK VLEIPYKGKD
LSMIVLLPNE IDGLQKLEEK LTAEKLMEWT SLQNMRETRV DLHLPRFKVE ESYDLKDTLR
TMGMVDIFNG DADLSGMTGS RGLVLSGVLH KAFVEVTEEG AEAAAATAVV GFGSSPTSTN
EEFHCNHPFL FFIRQNKTNS ILFYGRFSSP
```

G3HYQ1 (100%), 27,689.8 Da
14-3-3 protein sigma OS=Cricetulus griseus GN=I79_016192 PE=3 SV=1
6 exclusive unique peptides, 8 exclusive unique spectra, 9 total spectra, 51/248 amino acids (21% coverage)

```
MEKASLIQKA KLAEQAERYE DMAAFMKGAV EKGEELSCEE RNLLSVAYKN VVGGQRAAWR
VLSSIEQKSN EEGSEEKGPE VKEYREKVET ELRGVCDTVL GLLDSHLIKE AGEAGSRVFY
LKMKGDYYRY LAEVATGDDK KRIIDSARSA YQEAMDISKK EMPPTNPIRL GLALNFSVFH
YEIANSPEEA ISLAKTTFDE AMADLHTLNE DSYKDSTLIM QLLRDNLTLW TADSAGEEGG
EAPEEPQS
```

Fig.S4 Mass spectrometric identification of APMAP-interacting proteins. Peptides identified by LC-MS/MS after tryptic digestion of eight different APMAP-containing low and high molecular weight protein complexes (CP1-CP8) from selected SEC fractions resolved by native-PAGE (see Fig.3) are highlighted in yellow in the primary sequences. Modifications are highlighted in green.

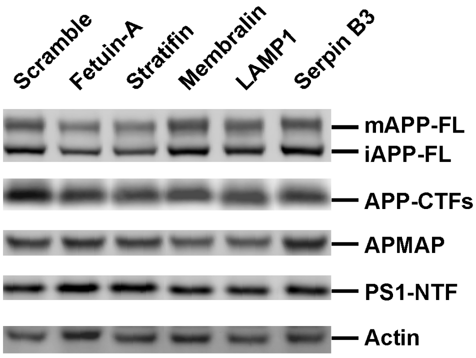

Fig.S5 Treatments with siRNAs targeting fetuin-A, serpinB3, stratifin, LAMP-1 and membralin do not affect APP-FL, APP-CTFs, or APMAP. The knockdown of the indicated APMAP-interacting proteins was performed as described in Fig.4 by small interfering RNA (siRNA) in HEK cells overexpressing APP bearing the Swedish mutation that causes early-onset familial Alzheimer's disease (HEK-APPSwe). Cells were treated for 3 days with siRNA duplexes (listed in the Materials and Methods section) complexed with Lipofectamine RNAiMax (Invitrogen). Whole cell extracts were prepared in HEPES buffer containing 1% NP40 and analyzed by Western blot for the siRNA protein targets APP-FL, APP-CTFs and APMAP. Cells transfected with the allstar control siRNA (scramble) served as negative controls while actin served as a protein loading control. APP-FL: APP full-length; APP-CTFs: APP-C-terminal fragments.

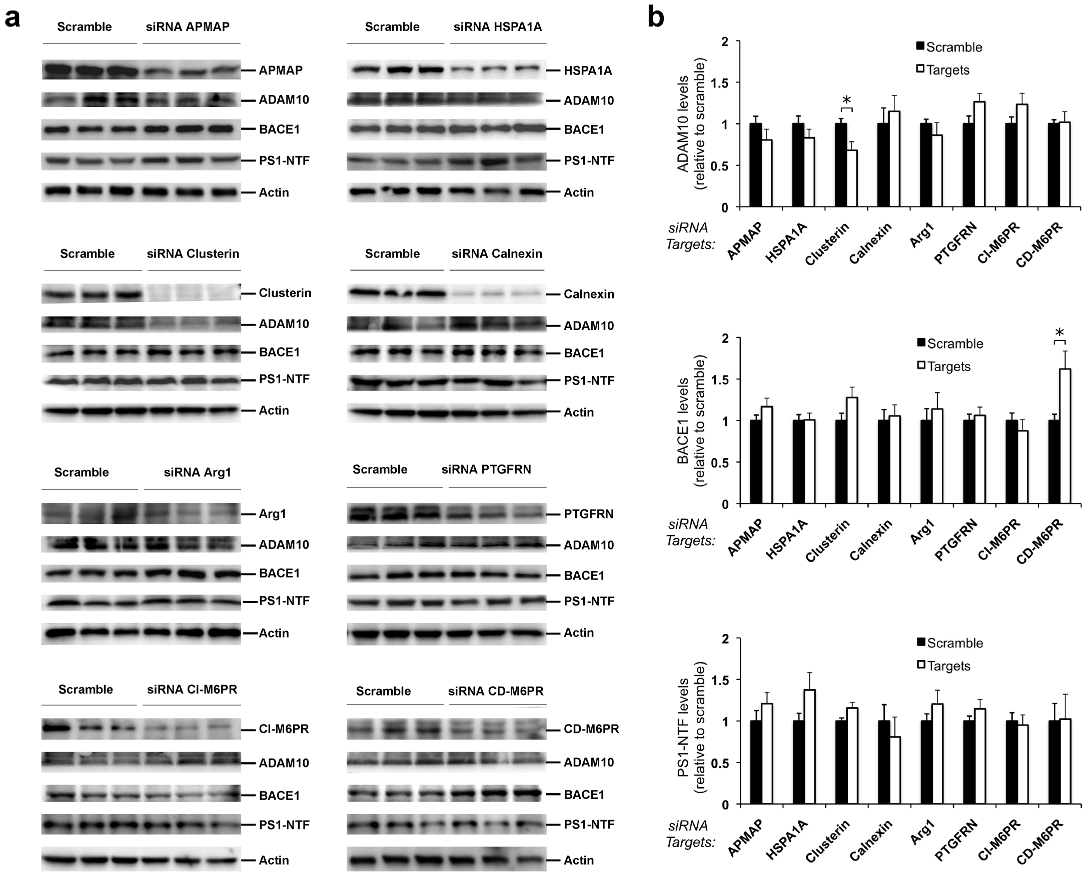

Fig.S6 Depletion of individual APMAP interacting proteins does not affect the level of α-, β- or γ-secretases. **a** The same whole-cell extracts as in Fig.4a, prepared from biological triplicates of HEK-APPSwe cells treated for three days with siRNA duplexes targeting the indicated APMAP-interacting proteins were analyzed by Western blot for the siRNA proteins targets, α-secretase ADAM10, β-secretase BACE1 and γ-secretase catalytic site protein presenilin-1 N-terminal fragment (PS1-NTF). Cells transfected with the allstar control siRNA (scramble) served as negative controls while actin served as a protein loading control. **b** Densitometric analysis of the ADAM10, BACE1 and PS1-NTF Western blot bands in (**a**). Student's *t*-test was applied for statistical analysis; significance is shown as the mean ± SEM, *P<0.05; ADAM10, BACE1 and PS1-NTF: n=3/group.

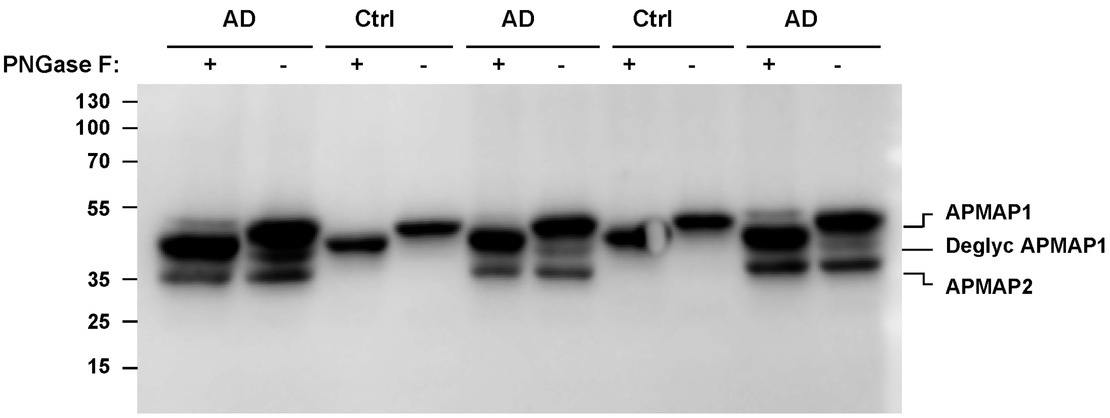

Fig.S7 N-linked glycosylation of APMAP1 and absence of glycosylation of APMAP2. The glycosylation profiles of APMAP1 and APMAP2 were estimated by Western blot analysis of denatured human cortical lysates of two control brains and three neuropathologically verified AD brains treated for one hour at 37°C in the presence (+) or absence (-) of Peptide-N-glycosidase F (PNGase F) to catalyze the cleavage of N-linked oligosaccharides. For APMAP1, the PNGase-dependent band shift (from APMAP1 to Deglyc APMAP1) reveals an N-linked glycosylation. For APMAP2, the absence of a band shift confirms an unglycosylated protein.

a

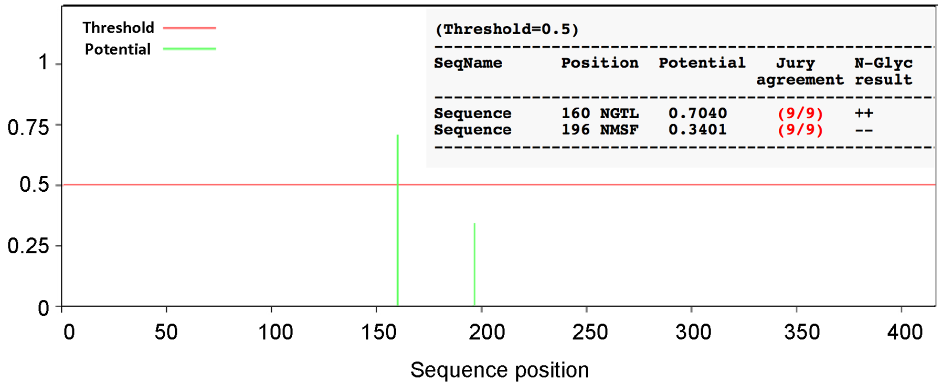

b

APMAP1:

MSEADGLRQRRPLRPQVVTDDDGQAPEAKDGSSFSGRVF**RVTFLMLAVSLTVPLLGAMMLL**ESPIDPQP
LSFKEPPLLLGVLHPNTKLRQAERLFENQLVGPESIAHIGDVMFTGTADGRVVKLENGEIETIARFGSG
PCKTRDDEPVCGRPLGIRAGP**N**GTLFVADAYKGLFEVNPWKREVKLLLSSETPIEGKNMSFVNDLTVTQ
DGRKIYFTDSSSKWQRRDYLLLVMEGTDDGRLLEYDTVTREVKVLLDQLRFPNGVQLSPAEDFVLVAET
TMARIRRVYVSGLMKGGADLFVENMPGFPDNIRPSSSGGYWVGMSTIRPNPGFSMLDFLSERPWIKRMI
FKLFSQETVMKFVPRYSLVLELSDSGAFRRSLHDPDGLVATYISEVHEHDGHLYLGSFRSPFLCRLSLQ
AV

c

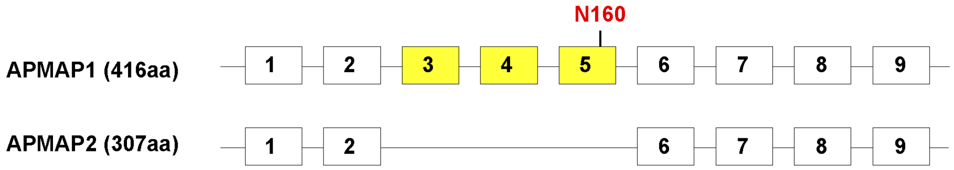

Fig.S8 APMAP1 predicted N-glycosylation at residue Asn160, while APMAP2 is unglycosylated. **a** Glycosylation site prediction in human APMAP1 with NetNGlyc 1.0 (http://www.cbs.dtu.dk/services/NetNGlyc/), by using artificial neural networks that analyzed the sequence context of Asn-Xaa-Ser/Thr. Asn160 was the only site with a predicted high probability N-glycosylation (potential > 0.5 and Jury agreement 9/9). **b** Primary sequence of human APMAP1 with the cytosolic N-terminal domain underlined, the transmembrane domain in bold and exons 3, 4 and 5 in yellow. The predicted N-glycoylated residue Asn160 on exon 5 is displayed in red. **c** The human alternative splicing variant APMAP2 lacks exons 3 and 4 and exon 5 containing the predicted glycosylation site Asn160.

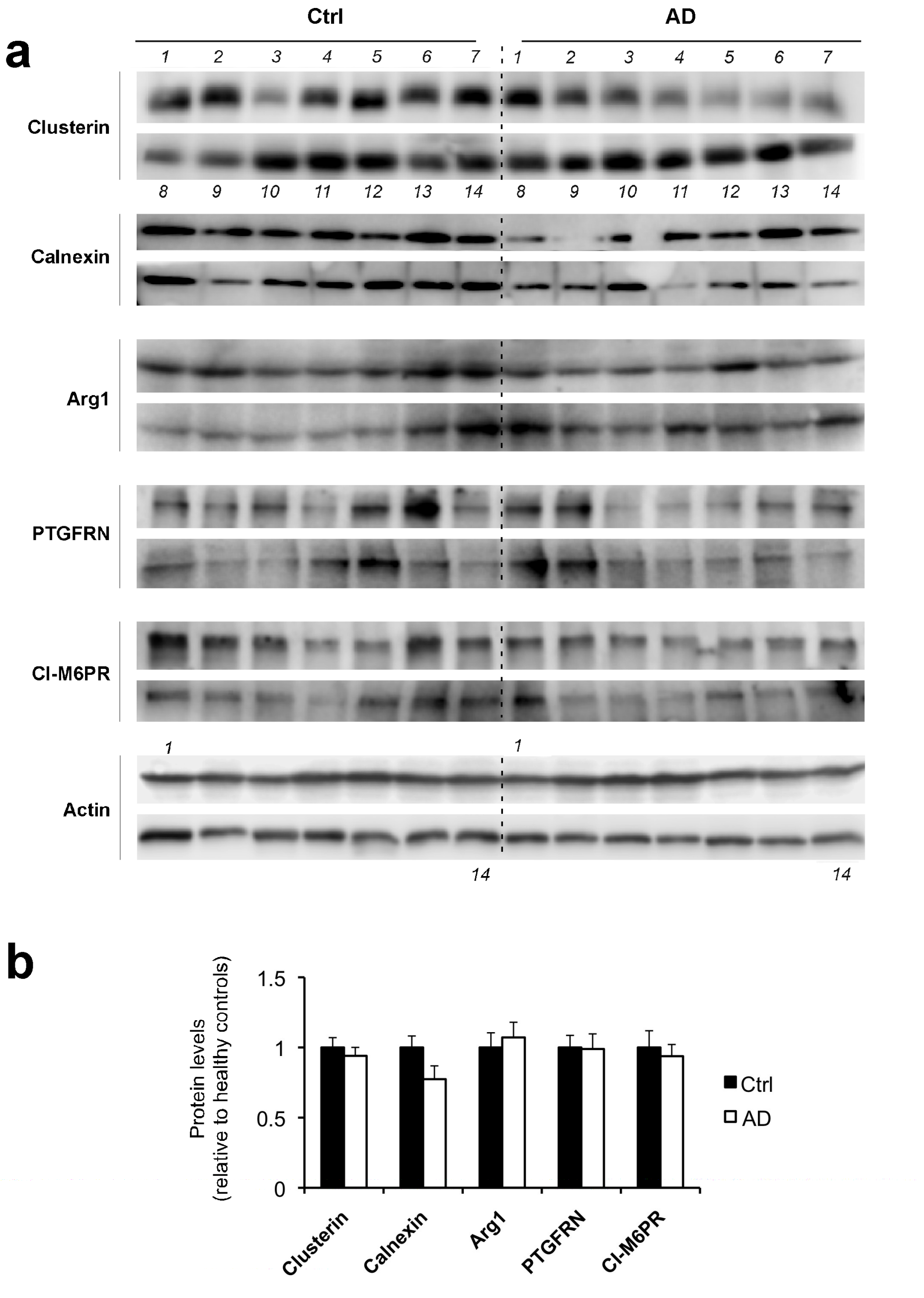

Fig.S9 The protein levels of the APMAP interactomers clusterin, calnexin, Arg1, PTGFRN and CI-M6PR are unchanged in AD brains. **a** The protein levels of the indicated APMAP interactomers were estimated by Western blot analysis in cortical lysates of 14 control brains and 14 neuropathologically verified AD brains (same samples as in Figure 5). **b** The densitometric analysis of the Western blot bands in (**a**) revealed no statistically relevant changes (Student's t-test) in the control and AD brains.
